# Cytobacts: Abundant and Diverse Vertically Seed-Transmitted Cultivation-Recalcitrant Intracellular Bacteria Ubiquitous to Vascular Plants

**DOI:** 10.3389/fmicb.2022.806222

**Published:** 2022-03-07

**Authors:** Pious Thomas, Thekepat P. Rajendran, Christopher M. M. Franco

**Affiliations:** ^1^Thomas Biotech & Cytobacts Centre for Biosciences, Bengaluru, India; ^2^Division of Biotechnology, ICAR-Indian Institute of Horticultural Research, Bengaluru, India; ^3^Research Information System for Developing Countries, India Habitat Centre, New Delhi, India; ^4^Department of Medical Biotechnology, College of Medicine & Public Health, Flinders University, Bedford Park, Adelaide, SA, Australia

**Keywords:** Brownian motion, endophytic bacterial diversity, metagenomics 16S, next-generation deep sequencing, plant cell biology, plant kingdom, plant tissue culture, uncultivable bacteria

## Abstract

We have recently described ‘Cytobacts’ as abundant intracellular endophytic bacteria inhabiting live plant cells based on the observations with callus and cell suspension cultures of grapevine and other plant species with the origin ascribable to field explants. In this study, we investigated the prevalence of such cytoplasmic bacterial associations in field plants across different taxa, their cultivability, and the extent of taxonomic diversity and explored the possibility of their embryo-mediated vertical transmission. Over 100 genera of field plants were surveyed for ‘Cytobacts’ through bright-field live-cell imaging as per our previous experience using fresh tissue sections from surface-sterilized shoot-tissues with parallel cultivation-based assessments. This revealed widespread cellular bacterial associations visualized as copious motile micro-particles in the cytoplasm with no or sparse colony forming units (CFU) from the tissue-homogenates indicating their general non-cultivability. Based on the ease of detection and the abundance of ‘Cytobacts’ in fresh tissue sections, the surveyed plants were empirically classified into three groups: (i) motile bacteria detected instantly in most cells; (ii) motility not so widely observed, but seen in some cells; and (iii) only occasional motile units observed, but abundant non-motile bacterial cells present. Microscopy versus 16S-rRNA V3–V4 amplicon profiling on shoot-tip tissues of four representative plants—tomato, watermelon, periwinkle, and maize—showed high bacterial abundance and taxonomic diversity (11–15 phyla) with the dominance of Proteobacteria followed by Firmicutes/Actinobacteria, and several other phyla in minor shares. The low CFU/absence of bacterial CFU from the tissue homogenates on standard bacteriological media endorsed their cultivation-recalcitrance. Intracellular bacterial colonization implied that the associated organisms are able to transmit vertically to the next generation through the seed-embryos. Microscopy and 16S-rRNA V3–V4 amplicon/metagenome profiling of mature embryos excised from fresh watermelon seeds revealed heavy embryo colonization by diverse bacteria with sparse or no CFU. Observations with grapevine fresh fruit-derived seeds and seed-embryos endorsed the vertical transmission by diverse cultivation-recalcitrant endophytic bacteria (CREB). By and large, Proteobacteria formed the major phylum in fresh seed-embryos with varying shares of diverse phyla. Thus, we document ‘Cytobacts’ comprising diverse and vertically transmissible CREBs as a ubiquitous phenomenon in vascular plants.

## Introduction

All plants and plant organs are known to harbor endophytic bacteria, the prokaryotes that inhabit plants internally without apparent adverse effects on the host (Hallmann et al., 1997; Kandel et al., 2017). Bacterial endophytes are known to be acquired by plants primarily from the rhizosphere, occasionally from the atmosphere, and also as seed-borne microbial communities (Hardoim et al., 2015; Walsh et al., 2021). Field plants show the highest population and diversity of endophytic bacteria in soil-embedded roots (Conn and Franco, 2004; Liu et al., 2017). Lower bacterial diversity commonly observed in root tissues relative to the rhizosphere has contributed to the assumption that plants selectively recruit a subset of their choice organisms from soil as endophytes (Compant et al., 2010, 2021; Hardoim et al., 2015). One major and initial route of plant entry of endophytic bacteria is through root hairs (Prieto et al., 2011; Compant et al., 2021). This is particularly applicable for seed-associated bacteria making their entry at seed germination from the spermosphere (Nelson, 2018). Once inside root the epidermis, the microorganisms find their way to the root vascular tissues traversing the parenchymatous cortex at which phase is mostly intracellular (Hallmann et al., 1997; Compant et al., 2005). As per the general understanding, the organisms move further upward through the vascular system and reach various plant parts displaying intercellular colonization (Compant et al., 2005, 2008, 2011; Kandel et al., 2017; Liu et al., 2017). Thus, the endophytic bacterial association is generally known to be intercellular or apoplastic (Sattelmacher, 2001; Hardoim et al., 2015; Liu et al., 2017). Plant acquisition of endophytes from the aerial parts through natural openings such as stomata, lenticels, and injuries incited by insects and other pests is also proposed, but in such cases too, the colonization is generally intercellular (Frank et al., 2017; Kandel et al., 2017).

We have recently documented abundant and diverse cytoplasmic/intracellular bacteria, termed ‘Cytobacts,’ in healthy plant cells as per microscopic observations including fluorescent *in situ* hybridization (FISH) targeting different classes of bacteria and 16S rRNA gene amplicon profiling on long-term actively maintained cell-suspension and callus cultures of grapevine and other plant species (Thomas and Franco, 2021). The ability to maintain *in vitro* cultures protected from external microorganisms offered the strong point in drawing the aforementioned conclusion contrary to the general practice of using *in situ* or *ex situ* plants for studying the endophytic microorganisms. Further, the organisms proved to be not amenable to cultivation which to some extent explained why this association went unnoticed so long. ‘Cytobacts’ were first described with banana shoot-tip tissues and *in vitro* cultures as conventionally uncultivable abundant bacteria colonizing the cytoplasm and also as adherents to the plasma membrane or organelles (Thomas and Reddy, 2013; Thomas and Sekhar, 2014). Subsequently they were documented with the field plants and *in vitro* cultures of papaya (Thomas et al., 2019). This conclusion on cytoplasmic bacteria was further strengthened with the isolated reports on intracellular bacteria documented in the meristem tissue of pine (Pirttilä et al., 2000), axenically grown pineapple and orchids (Esposito-Polesi et al., 2017), and ‘Bacteriosomes’ observed in axenic peach palm plants (de Almeida et al., 2009). The elucidation of ‘Cytobacts’ was facilitated through bright-field live-cell imaging displaying abundant motile micro-particles in the cytoplasm resembling the cells of common endophytic bacteria, supported by live bacterial staining using the DNA stain SYTO-9 (Thomas and Reddy, 2013; Thomas and Sekhar, 2014). Extension of this study to long-term actively maintained cell and callus cultures of grapevine and other plant species supported by fluorescence and confocal microscopy employing bacterial stains, FISH, and next-generation deep sequencing revealed cytoplasmic bacterial association as a wide-spread phenomenon for *in vitro* cultures across plant species (Thomas and Franco, 2021). An exploration on to the possible sources of these cytoplasmic bacteria in cultured plant cells indicated the prevalence of such intracellular bacteria in field-pants. In these microscopic studies, no bacteria could be detected in the intercellular regions to support the broad inference of apoplastic colonization by endophytic bacteria (Hallmann et al., 1997; Sattelmacher, 2001; Hardoim et al., 2015). Although there is substantial literature on endophytic bacterial associations covering their biology, diversity, functions in plants, and their potential exploitation for optimizing crop production, very few studies have provided microscopic data on tissue colonization, which too in most instances have targeted just the root system (White et al., 2014, 2018; Shehata et al., 2017). Most of these reports and reviews go with the general statements on endophytic bacterial colonization as intercellular and/or intracellular (Hardoim et al., 2015; Kandel et al., 2017; Liu et al., 2017).

The wide-spread cytoplasmic bacterial associations observed in cultured cells of different plant species *in vitro* and the occasional reports on intracellular bacteria cited above made it imperative to investigate the prevalence of such associations in field plants across diverse taxa. Cultivation *versus* microscopy-based studies on field plant tissues of banana earlier indicated that the associated organisms were normally uncultivable (Thomas and Sekhar, 2014). Scientific studies employing molecular tools such as 16S rRNA ribotyping; 16S rRNA gene amplicon profiling; or whole genome metagenomics on banana, grapevine, papaya, etc., have brought to light a huge diversity of uncultivable/cultivation-recalcitrant endophytic bacteria (CREB) (Thomas and Sekhar, 2017; Thomas et al., 2017, 2019). Generally, Proteobacteria formed the major phylum followed by Actinobacteria, Firmicutes, and Bacteroidetes, and several other phyla including some candidate phyla with no or sparse cultivable counterparts.

One major question with respect to the intracellular bacterial colonization pertained to how the organisms gain cytoplasmic entry throughout the plant system without damaging the cells, concurring with the widely accepted concept of plant recruitment of endophytic bacteria from soil or atmosphere. This becomes feasible in the case of embryo-mediated vertical transmission (Thomas and Sahu, 2021). Intracellular colonization implies that the organisms are able to move to the daughter cells/gametes through mitosis/meiosis and then to the embryo, which allows their distribution to all parts of the new plant similar to cell organelles. This aspect is best studied by analyzing the seed-embryos for any associated bacteria, taking care to exclude all no-embryonic seed-tissues. Seed transmission of bacterial endophytes is a topic of much recent interest suggesting the possibility of vertical microbial movement from one generation to the next (Nelson, 2018; Shahzad et al., 2018; White et al., 2019; Matsumoto et al., 2021). Seed-associated microbes could inhabit the seed internal tissues and/or the external tissues, and hence, the seed association does not essentially mean vertical transmission unless the organisms colonize the embryo *per se* (Thomas and Shaik, 2020). Most studies on seed transmission of endophytic bacteria have explored this aspect primarily employing surface-sterilized seeds (Khalaf and Raizada, 2016; Glassner et al., 2018; Nelson, 2018). In such studies, there is a possibility of carry-over of externally associated bacteria despite extensive surface-sterilization treatments. It is essential that the embryo be studied distinctly excluding the seed coat tissues to confirm the vertical transmission (Thomas and Sahu, 2021). The report on ‘Cytobacts’ as a widespread association in different plant tissues and as a general phenomenon in cell and callus cultures across plant species substantiated the possibility of their movement to the gametes, possible integration in the new embryos during fertilization, and the continued perpetuation in the new plant. This is substantiated by the report on pollen or ovule mediated-transmission of endophytic bacteria (Frank et al., 2017; Maniranjan et al., 2017).

Thus, the present study was taken up as a continuation of the observations on the prevalence of abundant and diverse endophytic bacteria in cell cultures of different plant species with their origin traced to the field explants (Thomas and Franco, 2021) and their possible vertical transmission (Thomas and Sahu, 2021). Further, we found bright-field live-tissue imaging as an easy and simple way to observe the intracellular bacteria (Thomas and Sekhar, 2014; Thomas and Franco, 2021), whereas the deep-sequencing or omics-based tools help in studying the diversity of conventionally uncultivable plant microbiome (Kaul et al., 2016; Matsumoto et al., 2021). The present study had the objectives of assessing the prevalence of intracellular bacteria in field plants across different functional and taxonomic categories, estimating the gross taxonomic diversity including CREB, and investigating the embryo-mediated vertical transmission.

## Materials and Methods

### General Experimental Design

Live cell bright-field microscopy imaging under high magnification (1000×) without stains or using low levels of Safranin/Grams crystal violet and micro-videography on microbial cell-motility was employed as a general tool for surveying different plant species for the cellular bacterial associations (Thomas and Sekhar, 2014; Thomas and Franco, 2021). A parallel cultivation-based assessment of bacterial CFU from surface-sterilized tissues was undertaken on representative plants. Further, a cultivation-independent determination of endophytic bacterial microbiome diversity adopting 16S rRNA gene V3–V4 taxonomic profiling of four selected/representative field plants was undertaken. In order to verify the possible embryo-mediated vertical transmission of endophytic bacteria, bacterial taxonomic profiling on clearly excisable embryos from mature watermelon seeds through 16S rRNA gene V3–V4 profiling and whole genome metagenome profiling was undertaken. Considering that grape cell cultures formed the main experimental material for the elucidation of intracellular bacteria in the earlier study (Thomas and Franco, 2021), bacterial taxonomic profiling on whole grape seeds gathered aseptically from ripe berries and the seed-embryos excised from hard grape seeds after alkali treatment were employed in 16S rRNA gene taxonomic profiling with parallel cultivation-based bacterial monitoring. As a prelude to this study, pure cultures of some bacterial strains isolated as endophytes, namely, *Klebsiella pneumonia* (medium motile rods), *Microbacterium esteraromaticum* (small motile rods), and *Micrococcus terrus* (motile cocci) were captured under bright-field microscopy for a comparison of their size, shape, and motility patterns to the motile micro-particles in tissue sections or the issue homogenates along with a common laboratory contaminant *Bacillus pumilus* (longer motile rods).

### Field Plants for Microscopic Observations

This involved about 100 genera of field-plants covering cereal, pulse, oilseed, fruit, vegetable, ornamental, medicinal, tuber and forage crops, grasses, weeds, and forest species (Table 1). The list included herbs, shrubs, climbers, and trees across annual and perennial species covering dicots and monocots mostly from India (Bengaluru, Karnataka State, or parts of neighboring Kerala and Tamil Nadu States) and some Australian plants (Adelaide, SA, Australia). Tissue segments from tender shoot or petiole tissues of field-grown plants were generally used. Wherever field plants were not accessible, seedlings/plants raised in glasshouse on soil-based medium were employed. Tissues were used after surface sterilization, which involved two initial rinses in autoclaved distilled water (ADW) containing 0.1% Tween-20 and 5–6 min sodium hypochlorite (2% available chlorine; Fisher Scientific, Mumbai, India) treatment with a change of disinfectant after 3 min, and final six rinses in sterile water. When tender tissues such as coleoptile or hypocotyl of seedlings was used, the duration of chemical treatment (3 min) or the strength of disinfectant was reduced (1% chlorine).

**TABLE 1:** List of plant species taken up for the bright-field microscopy-based survey for endophytic bacteria and an empirical classification of plants based on the ease of observing motile intracellular bacteria in fresh tissue sections.

	(i) Motile bacteria detected instantly in most cells				(ii) Motility not so widely observed in all cells, but seen in some cells with non-motile bacteria obvious in most cells				(iii) No obvious motile bacteria but abundant non-motile micro-particles corresponding to bacteria present		
	Genus/species	Category^#^	Tissue		Genus/species	Category^#^	Tissue		Genus/species	Category^#^	Tissue
1	*Acalypha hispida*	A	Petiole	1	*Allamanda cathartica*	P	Petiole	1	*Abelmoschus esculentus* (Supplementary Movie 12)	A	Petiole
2	*Amaranthus* sp.	A	Petiole	2	*Allium cepa*	A	Leaf base	2	*Anacardium occidentale*	P	Petiole
3	*Ananas comosus*	S	Crown	3	*Arabidopsis thaliana*	A	Hypocotyl	3	*Annona reticulata*	P	Petiole
4	*Aster patens*	A	Stem	4	*Averrhoa carambola*	P	Petiole	4	*Anthurium andraeanum*	S	Petiole
5	*Beta vulgaris*	A	Stem	5	*Begonia semperflorens-cultorum*	S	Petiole	5	*Araucaria excelsa* (Supplementary Movie 13)	P	Rachis
6	*Bougainvillea glabra*	P	Stem	6	*Brassica nigra*	A	Stem	6	*Azadirachta indica*	P	Petiole
7	*Brassica oleracea*	A	Midrib	7	*Carica papaya*	P	Petiole	7	*Casuarina equisetifolia*	P	Petiole
8	*Canna generalis*	S	Leaf base	8	*Cicer arietinum*	A	Hypocotyl	8	*Cosmos bipinnatus*	A	Petiole
9	*Capsicum annuum* (Supplementary Movie 06)	A	Petiole, Stem	9	*Citrus aurantifolia*	P	Petiole	9	*Cycas revoluta*	P	Rachis
10	*Catharanthus roseus*	S	Petiole	10	*Citrus pummelo*	P	Petiole	10	*Dracaena kaweesakii*	S	Leaf base
11	*Celosia argentea*	A	Stem	11	*Codiaeum variegatum*	P	Petiole	11	*Dypsis lutescens*	P	Rachis
12	*Centella asiatica*	A	Petiole	12	*Cynodon dactylon*	S	Stem	12	*Eupatorium odoratum*	S	Petiole
13	*Chlorophytum comosum*	S	Leaf base	13	*Cyprus rotundus*	S	Leaf base	13	*Garcinia indica*	P	Petiole
14	*Chrysanthemum indicum* (Supplementary Movie 09)	S	Stem, petiole	14	*Dendrobium* sp.	P	Leaf base	14	*Gladiolus palustris*	S	Leaf base
15	*Citrullus lanatus*	A	Stem	15	Duranta goldiana	P	Petiole	15	*Grevillea robusta*	P	Stem
16	*Colocasia esculenta*	S	Petiole	16	*Eleusine coracana*	A	Coleoptile	16	*Hibiscus rosa-sinensis*	P	Stem, petiole
17	*Coriandrum sativum*	A	Stem	17	*Geranium* sp.	S	Petiole	17	*Hylocereus undatus*	P	Stem
18	*Cucurbita moschata*	A	Stem	18	*Impatiens balsamina*	A	Stem	18	*Lagerstroemia speciosa*	P	Stem
19	*Dracaena braunii*	S	Leaf base	19	*Ipomoea indica*	S	Stem	19	*Lantana camera*	P	Stem
20	*Epipremnum aureum*	S	Petiole	20	*Ixora coccinea*	P	Petiole	20	*Lilium longiflorum*	S	Leaf
21	*Euphorbia pulcherrima*	S	Petiole	21	*Jasminum sambac*	S	Stem	21	*Mimosa pudica*	S	Stem
22	*Fragaria × ananassa*	S	Petiole	22	Leucaena leucocephala	S	Stem	22	*Mangifera indica*	P	Petiole
23	*Gerbera* sp.	A	Petiole	23	*Lonicera sempervirens*	S	Stem	23	*Maranta arundinacea*	S	Petiole
24	*Mentha × piperita*	A	Stem	24	Michelia champaca	P	Petiole	24	*Nephrolepis exaltata*	S	Rachis
25	*Mirabilis jalapa*	S	Stem	25	*Manihot esculenta*	S	Petiole	25	*Peltophorum pterocarpum*	P	Petiole
26	*Momordica charantia*	A	Stem	26	*Mussaenda erythrophylla*	S	Petiole	26	*Philodendron bipinnatifidum*	S	Leaf
27	*Mucuna bracteata*	S	Stem	27	*Oryza sativa* (Supplementary Movie 10)	A	Coleoptile	27	*Phyllostachys aurea*	S	Leaf base
28	*Musa* sp.	P	Shoot tip	28	*Oxalis* sp.	A	Petiole	28	*Polyalthia longifolia*	P	Stem
29	*Nicotiana tabacum*	A	Stem, Petiole	29	*Passiflora edulis*	P	Petiole	29	*Psidium guajava*	P	Petiole
30	*Ocimum sanctum*	S	Stem	30	*Polianthes tuberosa*	S	Leaf base	30	Ricinus communis	S	Petiole
31	*Pachystachys lutea*	S	Stem	31	*Punica granatum*	P	Petiole	31	*Spathiphyllum wallisii*	S	Leaf base
32	Pentas lanceolata	P	Stem	32	*Rosa indica*	P	Petiole	32	*Syzygium cumini*	P	Petiole
33	*Phaseolus vulgaris* (Supplementary Movie 08)	A	Petiole	33	*Sesbania drummondii*	S	Stem	33	*Syzygium samarangense*	P	Petiole
34	*Pisum sativum* (Supplementary Movie 07)	A	Petiole	34	*Strelitzia reginae*	S	Leaf base	34	*Tecoma grandiflora*	P	Petiole
35	*Raphanus sativus*	A	Petiole	35	*Triticum aestivum* (Supplementary Movie 11)	A	Coleoptile	35	*Terminalia catappa*	P	Petiole
36	*Salvia splendens*	A	Stem	36	*Vitis vinifera*	P	Petiole	36	*Thuja occidentalis*	P	Stem
37	*Solanum lycopersicum* (Supplementary Movie 05)	A	Stem, Petiole	37	*Zea mays*	A	Leaf base	37			
38	*Solanum melongena*	A	Petiole								
39	*Spinacia oleracea*	A	Petiole								
40	*Syngonium podophyllum*	A	Petiole								
41	*Tagetes patula*	A	Petiole								
42	*Zingiber officinale*	A	Petiole								
43	*Portulaca grandiflora*	S	Petiole								

*^#^Category: A, annual; P, perennial; S, semi-perennial/semi-annual.*

### Tissue Microscopy

Preparation of thin tissue sections using a Cryotome FSE (Thermo Fisher Scientific, Cheshire, United Kingdom) was initially considered for bight-field live-cell imaging on surface-sterilized tender shoots of grapevine ‘Flame Seedless.’ Cryo-sections, however, showed tight adhesion to the glass slide due to the cryo-gel. Further, the cryo-sections mounted in water did not show any micro-particle motility. The tissue sections also failed to take the live bacterial stain SYTO-9 suggesting cryo-gel imparted inhibition of DNA staining.

Thin free-hand tissue sections prepared from tender shoot tissues—terminal stem tissue, petiole, leaf base, or rachis—were employed for bright-field cell imaging. Surface sterilized tissues were sectioned over a sterile glass slide (washed in soap solution, wiped with 70% ethanol and flamed twice with absolute alcohol dip) using a stainless razor blade (Vidyut Super-Max, Thane, India), and 4–5 thin sections (∼30–50 μm) were transferred to 0.2 μm filtered double-autoclaved distilled water (FDW) on a fresh glass slide. After two rinses in FDW, they were observed directly in FDW or in 0.001% safranin at 1000× under oil immersion using Leica DM2000 microscope (Leica Biosystems, Wetzlar, Germany) as described elsewhere (Thomas and Sekhar, 2014).

### Assessing Cultivable Endophytic Bacteria in Shoot-Tip Tissues

Shoot-tip tissues from field plants were surface-sterilized as above and then treated with 2.0% Na_2_S_2_O_3_ for 10 min to remove any residual toxic chloramines. After three rinses in sterile distilled water (SDW), the tissues were aseptically homogenized with a micro-pestle (1.5 ml tube) and the tissue homogenate (TH; 100 mg ml^–1^ FDW) was spread on nutrient agar (NA) through the SP-SDS method using 20-μl lots (Thomas et al., 2015) employing four replications. Prior to the TH preparation, the last two wash solutions post-surface sterilization (200 μl) were plated on NA as per spotting-and-tilt-spreading (Thomas et al., 2012), and the CFU estimates for TH were accepted after ensuring the effectiveness of surface sterilization for 2–4 days.

### 16S rRNA Gene V3–V4 Amplicon Profiling on Field Samples

Four plant species, namely tomato (*Solanum lycopersicum*), watermelon (*Citrullus lanatus*), periwinkle (*Catharanthus roseus*), and maize (*Zea mays*) were selected as representatives for cultivation-independent bacterial diversity assessment adopting 16S rRNA gene-V3–V4 amplicon profiling. Tomato, watermelon, and maize are common annual crops accessible the world over, the former two representing dicots and the latter, a monocot. The first two also formed the candidates for seed and embryo bacterial transmission studies (Thomas and Shaik, 2020; Thomas and Sahu, 2021). Periwinkle, a semi-perennial shrub, formed the source of cell cultures employed for the elucidation of intracellular bacteria (Thomas and Franco, 2021) along with grapevine which was covered in detail earlier (Thomas et al., 2017).

A pooled tissue sample was drawn from the field shoot-tip tissues (25–50 g) of 1- to 2-month-old seed-derived field plants except in perennial periwinkle, and about 5 g tender tissues were surface-sterilized as above. To ensure effective tissue surface disinfection, the wash solutions were plated on NA and monitored for CFU for a week during which the tissue samples were stored at −20°C. After a week, the samples were thawed and homogenized in a mortar, and the TH (1 g FDW in 10 ml) was incubated at 4°C for 2 h to allow the larger particles to settle down. The tissue homogenates were tested for the share of cultivable bacteria through SP-SDS plating of decimal dilutions on NA and trypticase soy agar (TSA). DNA was extracted from the upper 3–4 ml clearer sample after high speed pelleting (18,000 × *g*) employing the Qiagen DNeasy PowerFood Microbial Kit (Qiagen GmbH, Hilden Germany; Cat. No. 21000-100) as per the extended lysis protocol. After preliminary quality and quantity assessments, one pooled DNA sample from three replications each were submitted to M/s Eurofins Genomics, Bengaluru^1^ for 16S rRNA V3–V4 taxonomic profiling.

16S rRNA gene amplicon libraries were prepared at M/s Eurofins Genomics targeting the V3–V4 hypervariable region as per the standard Illumina 16S Metagenomic Sequencing Library preparation protocol. Library sequencing on Illumina MiSeq platform (2 × 300 bp), quality filtrations, stitching, and QIIME (Quantitative Insights Into Microbial Ecology) bioinformatics analysis were done as described elsewhere (Thomas and Sekhar, 2017). Two rounds of QIIME analyses were undertaken as per Thomas and Sekhar (2017), the first round on the cleaned up data and the second round on the filtered data excluding sequences that corresponded to plastid and mitochondria 16S rRNA identified in the first round. Taxonomic assignments were made with Greengenes as the reference database.

### Cultivation Versus Microscopy-Based Assessment of Endophytic Bacteria in Watermelon Seed-Embryos

Seed-embryos of watermelon (cv. Arka Manik) were employed to assess the prospect of vertical transmission of bacterial endophytes considering the feasibility of clear excision of embryos excluding the seed external tissues. Fruits weighing about 3–4 kg after repeated surface wipes with 10% Domex were used as the source of fresh seeds. Seeds (50 nos) were extracted from the middle part of the fruits in a laminar air-flow (LAF), shaken vigorously in 0.01% Tween-20, rinsed thrice in SDW, treated with 70% ethanol (1 min), and air-dried over sterile tissue-paper. Twenty seeds each were collected per fruit and decoated aseptically using a sterile nail cutter. The excised embryos were treated with 90% ethanol (60 s) and then NaOCl (4% chlorine) for 5 min followed by six rinses in SDW, soaked in 2.0% Na_2_S_2_O_3_ for 10 min and then rinsed thrice in SDW with the plating of 400 μl of the last wash solution. Surface-sterilized seed-embryos were homogenized in a mortar employing 1 ml FDW per embryo which yielded a milky suspension. Original embryo-homogenate and five decimal dilutions were assessed for cultivable bacteria through SP-SDS using 20 μl lots (Thomas et al., 2015) on four replicate TSA plates while the 10^0^–10^–2^ lots were assessed on NA through spotting-and-tilt-spreading (SATS) (Thomas et al., 2012) and observed for CFU for 2 weeks at 30°C. Altogether, 10 fruits were studied as the source of seed-embryos in different batches.

### 16S rRNA Gene V3–V4 Amplicon Profiling of Watermelon Seed-Embryos

The embryo homogenates from the above 10 fresh fruit-derived seeds were stored at −20°C while waiting for the results from cultivation-based assessments. The homogenate from five seed lots which did show any cultivable bacteria for 1–2 weeks were used for the cultivation-independent analysis to avoid the over-representation of OTUs from easily cultivable organisms. The thawed embryo homogenates were pooled and incubated at 4°C for 2 h for the larger particles to settle down. The upper clearer 4 ml suspension with abundant microscopic motile particles was pooled and used for microbial DNA isolation after pelleting at 18,000 × *g* for 2 min. The supernatant part which showed some motile micro-particles was re-spun twice in a fresh tube. The pooled pellet from the three spins was used for DNA extraction employing (i) PowerFood Microbial DNA isolation (PF) kit (Laboratories Inc., Carlsbad, CA, United States) with extended 10 min at 70°C before the bead-beating step, or (ii) AxyPrep bacterial DNA isolation (AP) kit (Cat #: AP-MN-BT-GDNA-50; Axygen Biosciences, Union City, CA, United States). After preliminary quantity and quality assessments, the embryo-derived DNA pooled from different fruit sources were pooled and submitted to M/s Xcelris Labs Ltd. (Ahmedabad, India)^2^ for 16S rRNA gene V3–V4 taxonomic profiling (sample IDs MG09 and MG10, respectively, for PF and AP kits).

16S rRNA gene V3–V4 region amplicon library preparation, library sequencing, quality filtrations, stitching, and two rounds of QIIME bioinformatics analysis were done as described above. Further, the 16S V3–V4 reads were used for functional analysis through PICRUSt (Langille et al., 2013) as described elsewhere (Thomas et al., 2019).

### Whole Genome Metagenome Taxonomic and Functional Profiling of Watermelon Seed-Embryos

Whole genome metagenome (WMG) analysis involved taxonomic and functional profiling using the −20°C stored embryo-homogenate from five fruits (original 50 seed-embryos). The pooled embryo-homogenate was used directly for DNA extraction employing a PF kit without separating the upper clearer portion. After preliminary quality and quantity assessments, the DNA sample was submitted to M/s SciGenom Labs Private Limited, Cochin, India^3^, for NGS-mediated WMG profiling. This involved the standard Illumina protocol including genomic DNA fragmentation, adenylation, adapter ligation, PCR amplification, library preparation, size distribution analysis, and library sequencing on the Illumina HiSeq with 2 × 250 bp high-quality paired-end (PE) reads as described earlier for grapevine (Thomas et al., 2017). After read quality checks and data processing, metagenome assembly was carried out *de novo* using the Ray-Meta program and taxonomy tree construction was done using the MEGAN software. Functional annotation of all the contigs were carried out by SEED classification employing MEGAN software from DIAMOND BLASTX results. KEGG pathway analysis was performed for each contig sequences by assigning KEGG Orthology (KO) numbers obtained from known reference hits. Further, enzyme and pathway information were assigned to contigs based on KO.

### Assessing Seed and Seed-Embryo Bacterial Microbiome in Grapevine

Since grape (*Vitis vinifera* L.) cell cultures formed the primary tool for the elucidation of cytoplasmic bacterial associations in the previous study (Thomas and Franco, 2021), grape seeds/embryos formed the candidates to assess the extent of embryo-mediated vertical bacterial transmission and the comparative assessment of bacterial diversity in seeds *versus* seed-embryos. Seeds were collected aseptically from ripe berries and the seed-embryos were excised as described below.

#### Aseptic Collection of Mature Grape Seeds

Ripe fruits of ‘Red Globe’ (RG) imported from California, United States, to Bengaluru, India, and the locally grown ‘Bangalore Blue’ (BB) were collected from local home groceries. Bold berries (25 nos) from a single bunch with no external injuries or blemishes were gathered retaining 2–4-mm stalk-bits. To assess initial external microbial load, berries were washed five times in SDW with wash solution monitoring by spotting (10 × 1-μl lots) on NA. Berries were surface-sterilized using 90% ethanol (1 min) and NaOCl (4% available chlorine) for 10 min followed by six SDW washes and wash solution monitoring. After removing the stalk-bits, berries were opened in a LAF cabinet, and the seeds were collected. After blotting dry the seeds of any residual fruit pulp using sterile tissue paper, seeds were rinsed six times in FDW. One seed each from 25 berries was pooled, weighed aseptically (1.35 g for ‘RG’ and 1.45 g for ‘BB’ for 25 seeds) and then crushed in a mortar followed by extended manual grinding (15 min) using 1 ml FDW per 100 mg seed yielding a thick milky solution with black particles. The upper part of the suspension after 1 h at 4°C was used for seed DNA isolation.

#### Aseptic Collection of Seed-Embryos From Mature Grape Seeds

Fifty seeds each of ‘RG’ and ‘BB’ collected from bold and ripe berries in the above experiment were used for this. Mature grape seeds proved very hard to open manually. Based on pre-trials, seeds were soaked in the identified seed softening solution (20% NaOH in 0.1% Tween-20) for 18–24 h at room temperature (24°C–26°C), washed 10 times in SDW, scrub-dried on tissue paper, and then de-coated aseptically with the help of a pair of forceps and surgical blade. The whole and broken embryos were gathered, weighed aseptically, and washed 10 times in FDW during which most of the perisperm membrane got dislodged. The wash solutions were monitored on NA and TSA and the seed-embryos were stored at −20°C. Two weeks later, with no CFU from wash solutions and embryo imprints on NA and TSA, the seed-embryos (280 mg for ‘RG’ and 300 mg for ‘BB’ mg) were thawed in FDW, cleared of all floating particles and the perisperm tissue, and washed recurrently in FDW (with wash solution monitoring) followed by grinding to a fine slurry in a mortar (50 mg ml^–1^ FDW). The embryo homogenate was assessed for any cultivable bacteria through SP-SDS on NA and SATS on TSA for 1 week before proceeding to 16S rRNA taxonomic profiling.

#### Microbial DNA Extraction and 16S rRNA Gene V3–V4 Amplicon Profiling

The supernatants from the seed tissue homogenates of ‘RG’ and ‘BB’ after standing 1 h at 4°C (to allow the settling of black seed-coat particles) were used for the whole seed DNA extraction while the embryo-homogenates was used wholly for DNA isolation soon after preparation. After 4 min of spinning (12,000 × *g* in 1.8 ml × 2 columns) the seed and embryo homogenates gave thick pellets with cloudy supernatants. The supernatant part was collected, re-spun twice, and then pooled. The combined pellet was used for microbial DNA isolation using Qiagen DNeasy PowerFood Microbial Kit as per the outlined extended protocol with the final DNA elution in a 60-μl buffer. After preliminary quality and quantity assessments, the DNA samples were taken up for 16S rRNA gene V3–V4 profiling as per the standard Illumina protocol at M/s. Eurofins Genomics India, Bengaluru^1^ (see text footnote 1) with two rounds of QIIME analysis to remove the interfering plant chloroplast and mitochondrial sequences.

### Documentation, Data Analysis, and Accession Numbers

The field plants were observed mainly though bright-field live cell imaging on fresh tissue sections from surface-sterilized tissues, and the documentation involved mainly micro-videography under 100× oil immersion objective (effective 1000× magnification). The videos were captured in ‘avi’ format and converted to ‘mp4’ with the help of WinX HD Video Converter Deluxe 2018 Trial Version. Parallel cultivation-based CFU assessments were made on the shoot-tips of common plant species through SP-SDS. Analysis of 16S V3–V4 sequence data involved two rounds of QIIME to exclude the plant chloroplast and mitochondrial and originally unassigned sequences while the WMG data analysis was done through MEGAN. Only one bio-replicate sample was employed in different experiments keeping the primary objective of assessing whether the bacterial diversity was small or large in order to substantiate the microscopic observations and to serve as guide for future experiments.

The 16S V3–V4 NGS data generated on the shoot-tip tissues of four plant species have been deposited with NCBI under the project “Shoot- tip endophytic bacterial microbiome” with the Bioproject ID PRJNA705572, Bio-sample acc. nos. SAMN18092256 (tomato), SAMN18092258 (watermelon), SAMN18092402 (periwinkle) and SAMN18092404 (maize), NGS data for watermelon seed and embryo samples under the project title “Cucurbit Seed, Embryo & Seedling Microbiome” with the Bioproject ID PRJNA564696, Bio-sample acc. nos. SAMN12726312 for MG-09 and SAMN12726313 for MG.10. For grape seed and seed-embryos, the bacterial microbiome NGS data has been deposited under the Bioproject ID PRJNA701136, Bio-sample acc. nos. SAMN17848617 for EG-Gr.MG01 (RG-Seed), SAMN17848618 for EG-Gr.MG02 (BB-Seed), SAMN17848891 for EG-Gr.MG03 (RG-Embryo), and SAMN17848989 for EG-Gr.MG04 (BB-Embryo).

## Results

### Preliminary Microscopic Observations

Pure cultures of different bacteria isolated as endophytes were observed under bright-field microscopy, and the captured videos for some representative organisms—*Klebsiella pneumoniae*, *Microbacterium esteraromaticum, Micrococcus terrus*—have been provided to serve as a reference to assess the similarity of the motile micro-particles in the tissue sections to the bacterial cells in terms of size, shape, and motility patterns (Supplementary Movies 01–03) along with *Bacillus pumilus* (Supplementary Movie 04) isolated as external contaminant. Initial attempts employing cryo-microtome sections from grape shoot and petiole tissues did not work satisfactorily due to the tight adhesion of tissue sections to the glass slide and no micro-particle motility observed under bright-field. Live microscopic imaging on thin fresh tissue sections from tender shoots or petioles (∼30–50 μm) offered the best means to detect cellular bacteria directly or with safranin (0.005%).

### Bright-Field Live Microscopy on Diverse Field-Plants

Microscopic observations on fresh tissue sections derived from field gown plants, covering >100 genera/species of vascular plants, revealed cytoplasmic bacteria ‘Cytobacts’ readily in most cases. The ease with which motile bacterial cells were detected in tissue sections showed some variation (Table 1). Thus, three distinct plant categories were observed: (i) motile bacteria detected instantly in most cells, as documented with tomato, sweet pepper, hot pepper, egg-plant, garden peas, cowpea, French bean, coriander, amaranth, chrysanthemum, marigold, gerbera, crossandra, centella, money plant, and pineapple (Supplementary Movies 05–09); (ii) motility not so widely observed, but seen in some cells with non-motile bacteria obvious in most cells (e.g., rice, wheat, grape, citrus, etc.; Supplementary Movies 10, 11); and (iii) no or occasional motile bacteria observed but abundant non-motile micro-particles corresponding to bacteria present (e.g., hibiscus, okra, mango, pine, etc.; Supplementary Movies 12, 13). This may be an empirical list, but the observations explicitly indicated the ubiquitous presence of cellular bacteria across plant species/genera. Intracellular bacterial motility did not appear dynamic always with the active particle motility obvious in all plant hosts and across all host cells in a microscopy field. Cells exhibiting intact structural organization displayed bacteria aligning along the plasma membrane or adhering to organelles. The detection of ‘Cytobacts’ was enabled with the motility behavior for which cell disruption appeared a necessity. Bacterial motility was perhaps induced with the disruption of the cell cytoskeleton at tissue preparation, the vulnerability to which apparently varied with the plant species, tissue type, tissue age, and other factors.

**FIGURE 1:**
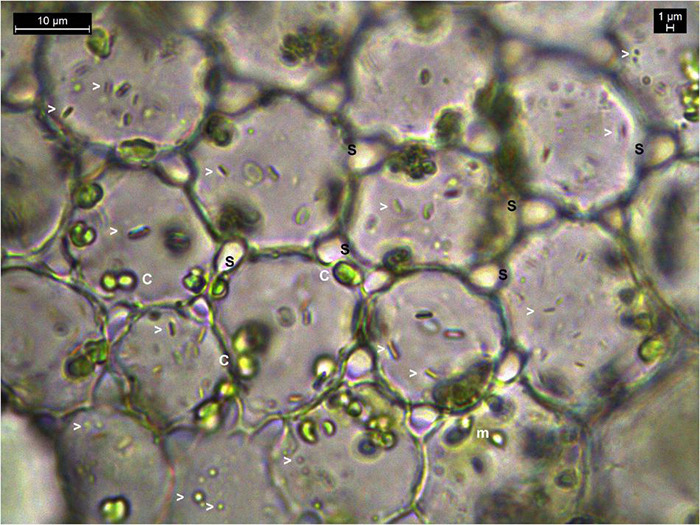
Bright-field microscopy on thin tissue section of water lettuce petiole (1000×). Tissue section showing abundant bacteria in the intracellular region. Arrowheads indicate bacterial cells; c, chloroplasts; m, mitochondria; s, inter-space between cells where no bacteria are observed.

Another noteworthy observation during microscopic explorations on tissue sections was the absence of obvious intercellular spaces or the bacterial cells in between the cells as generally reported for endophytic bacteria (Supplementary Figure 1 and Supplementary Movies 05–13). On the other hand, a direct snap shot of a bright field image with bacterial staining employing dilute crystal violet or safranin, or even without any stains, showed abundant intracellular bacteria in some instances (Figure 1). Even in cases where some intercellular spaces were noticed, no bacteria were observed in those regions, and the amount of microparticles seen inside the cells far outnumbered any possible inhabitants in the intercellular region.

### Cultivation- Versus Microscopy-Based Assessment of Endophytic Bacteria in Field Plant Shoot-Tip Tissues

Tissue homogenate from surface-sterilized field tissues of glasshouse or field grown plants yielded no colony forming units (CFU) on NA for 55% of 40 plant genera explored through the cultivation-based approach while the rest showed a few CFU (10^3^ g^–1^ tissue in 15%, 10^4^ in 20%, and 10^5^ in 10%) with one to five colony morphotypes for a plant species. This CFU appeared far too low compared with the abundant bacterial cells as documented earlier with the shoot-tip tissue sections, or observed with the tissue homogenates (Supplementary Movie 14), indicating the general non-cultivability of associated bacteria.

### Cultivation Versus 16S rRNA Gene V3–V4 Amplicon Profiling of Shoot-Tip Tissues of Representative Plants

Tissue samples from the four plant samples—tomato, watermelon, periwinkle, and maize—showed low CFU (10^3^–10^4^ g^–1^ tissue) with one to four different colony types during the 1 week observation period. Considering that our interest was merely to get an estimate of cultivable organisms relative to the bacterial cells observed during microscopy or molecular based diversity analysis, the organisms were not taken up for identification or further characterization. The DNA yields appeared low (7.3 ng μl^–1^ in maize to 11 ng μl^–1^ in tomato), yet they yielded clear PCR amplicons and good quality amplicon libraries with 0.14–0.17 million reads (Supplementary Table 1). QIIME round-1 analysis showed a large share of reads corresponding to chloroplasts and mitochondria (46.3% in periwinkle to 62.6% in maize). QIIME round-2 taxonomic analysis excluding the plant reads showed OTUs in the range of 117 (periwinkle) to 179 (maize) with Shannon alpha diversity indices in the range of 2.04 (watermelon) to 3.80 (maize).

#### Taxonomic Diversity

**FIGURE 2:**
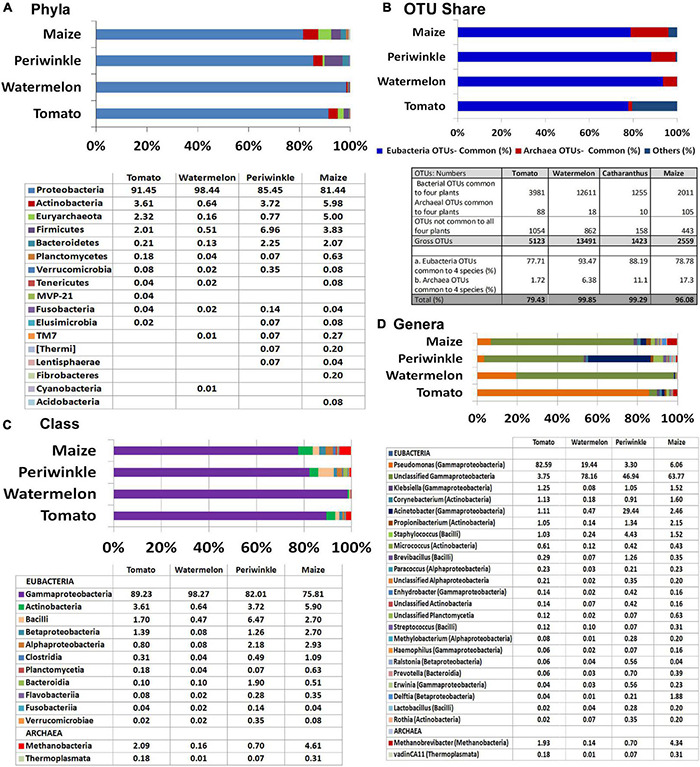
Illumina MiSeq NGS-based bacterial diversity analysis on surface-sterilized shoot-tip tissues of four plant species. 16S rRNA gene V3–V4 region profiling of tomato, watermelon, periwinkle, and maize showing **(A)** distribution of OTUs under different phyla. **(B)** OTU shares common to different plant species. **(C)** Major classes and **(D)** major genera.

Majority of the OTUs belonged to Eubacteria (95% in maize to 99.8% in watermelon) and the rest to Archaea, the latter appearing noteworthy for maize (5%) and tomato (2.3%). Assessing the eubacterial diversity, 16 phyla were documented across the four plant species with seven common phyla (Proteobacteria, Actinobacteria, Firmicutes, Bacteroidetes, Planctomycetes, Verrucomicrobia, and Fusobacteria) (Figure 2A). Proteobacteria formed the major component in all samples (81.4%–98.4%). Actinobacteria (0.64%–5.98%), Firmicutes (0.5%–3.78%), and Bacteroidetes (0.1%–2.25%) constituted the other notable phyla while the rest constituted very low shares. Archaeal OTUs were confined to one phylum, Euryarchaeota.

Overall, 307 OTU categories were observed across the four plant species including 116 in tomato, 142 in watermelon, 117 in periwinkle, and 179 in maize. The four plant species showed 34 common eubacterial OTU categories constituting 77.7%–93.5% of the total OTUs and three Archaea forming 1.7%–17.3% OTUs, both together forming 79.5%–99.9% of the OTUs (Figure 2B). Thus, besides a high bacterial diversity, the four plant species also displayed considerable taxonomic similarity indicating a core microbiome across the species. The limited CFU observed during cultivation-based study versus the documented high diversity suggested the organisms were largely uncultivable.

At the Class level, γ-Proteobacteria formed the major constituent in all the plant samples: 75.8% in maize to 98.3% in watermelon (Figure 2C). The next major classes included Actinobacteria followed by Bacilli for tomato, watermelon, and maize while periwinkle had more of Bacilli followed by Actinobacteria. Tomato and maize displayed a notable share of Methanobacteria under phylum Euryarchaeota contrary to the minor shares of this in other plant species. Grossly, tomato harbored 20 classes with watermelon, periwinkle, and maize showing 17, 18, and 26 constituents, respectively, with 13 classes common for the four samples. Pseudomonadaceae formed the major family in tomato (83.3%) while others had Moraxellaceae in the lead (65.5%, 69.3%, and 56.2% for watermelon, periwinkle, and maize, respectively). The four plant species showed 56, 42, 48, and 69, constituents, respectively, although many of these were present in very small amounts.

At the Genus level, *Pseudomonas* formed the largest single constituent in tomato (82.6%) while the other three samples showed undefined Moraxellaceae as the largest constituent along with a notable share of unclassified *Pseudomonadaceae* (Figure 2D and Supplementary Dataset 1). Periwinkle had *Acinetobacter* (Moraxellaceae) as the second major genus. Tomato showed OTU distribution under 76 eubacterial genera. The respective values for watermelon, periwinkle, and maize were 58, 63, and 87 with 27 genera common to the four species albeit the OTU shares were quite negligible in some instances. It was also striking to note six Archael taxa (*Methanobrevibacter, vadinCA11, Unclassified Methanobacteriaceae, Natronococcus, Methanosphaera*, and *Methanoplanus*) across the four plant species with the first two taxa observed in all of them. *Methanobrevibacter* constituted a major component in maize (4.4%) and tomato (1.9%). The observations here endorsed the prevalence of huge taxonomic diversity of Eubacteria and some amount of Archaea internally in field shoot-tip tissues in agreement with the abundant intracellular bacteria observed during microscopy.

#### PICRUSt Functional Analysis

**FIGURE 3:**
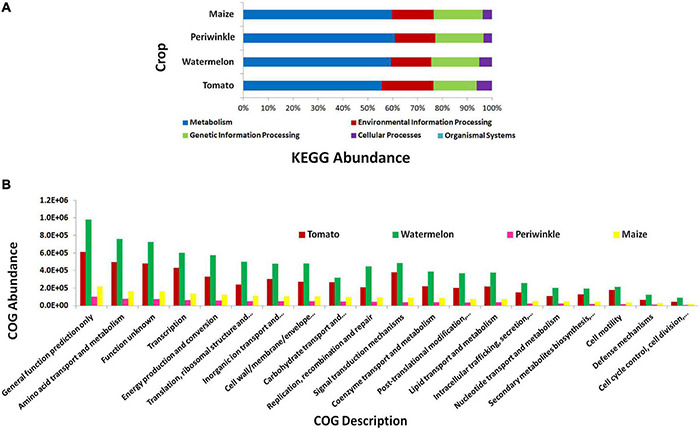
PICRUSt functional analysis on 16S rRNA V3–V4 region profiles with Illumina MiSeq NGS platform on different plant species, tomato, watermelon, periwinkle/*Catharanthus*, and maize. **(A)** KEGG Level 1 functional analysis and **(B)** COG Level 1 functions.

KEGG pathway analysis showed the microorganisms largely involved in metabolic pathways followed by environmental information processing and genetic information processing (Figure 3A). COG description indicated diverse but similar functional profiles for the endophytic bacterial biome in different plant species with general functional prediction and amino acid metabolism on the top (Figure 3B). Other functional roles involved transcription, energy production and conversion, signal transduction mechanisms, inorganic ion transport and metabolism, cell wall/membrane/envelope biogenesis, translation, ribosomal structure and biogenesis, and others. Thus, the organisms appeared to be involved potentially in various plant cellular processes.

### Cultivation- and Microscopy-Based Assessment of Endophytic Bacteria in Watermelon Seed-Embryos

The hard rind of watermelon fruits facilitated the gathering of seeds aseptically after surface sterilization, thus the isolation of seeds without external exposure (Supplementary Figure 2). Seed-embryos could be excised distinctly from air-dried seeds excluding the seed coat tissues with the help of a nail cutter. During NaOCl treatment, the membranous perisperm tissue covering the embryo got released leaving aside the clean seed-embryos. The embryo wash solutions post ethanol treatment of seeds and of embryos with NaOCl did not show any CFU on NA ensuring that no organisms external to the embryo were entering the processing line. The embryo homogenate—original stock and decimal dilutions—did not show any CFU on NA or TSA with both SATS and SP-SDS spreading procedures during the 1–2 weeks of observation. Monitoring the seeds from the subsequent fruits showed very few CFU from the original, 10^1^ or 10^2^ samples in some instances during the 1- to 2-week observation period, but none in others (Supplementary Figure 3).

The occasional CFU that developed from tissue homogenates after 1–2 weeks did not appear proportional to the dilution series, and it was translated to 10^1^ to 10^3^ CFU per embryo with just one to three colony morphotypes. These random CFUs proved too negligible considering the abundant motile bacterial cells observed during microscopic observations on the tissue homogenate (Supplementary Movie 15). This was particularly so considering that it warranted the use of 1:10 or 1:100 dilutions for microscopic observations since the original homogenate (1 embryo ml^–1^ FDW) was too dense for microscopy mounting. The observations indicated a huge bacterial presence in the embryo tissues considering that the 1:100 dilution barely had 10-μg tissues in a 20-μl sample loaded and that the microscopy field view covered only a negligible area of the homogenate spread. Here again, the organisms were not taken up for identification or further characterization since that was not a priority in this study.

### 16S rRNA Gene-Based Taxonomic Profiling on Watermelon Seed Embryos

**FIGURE 4:**
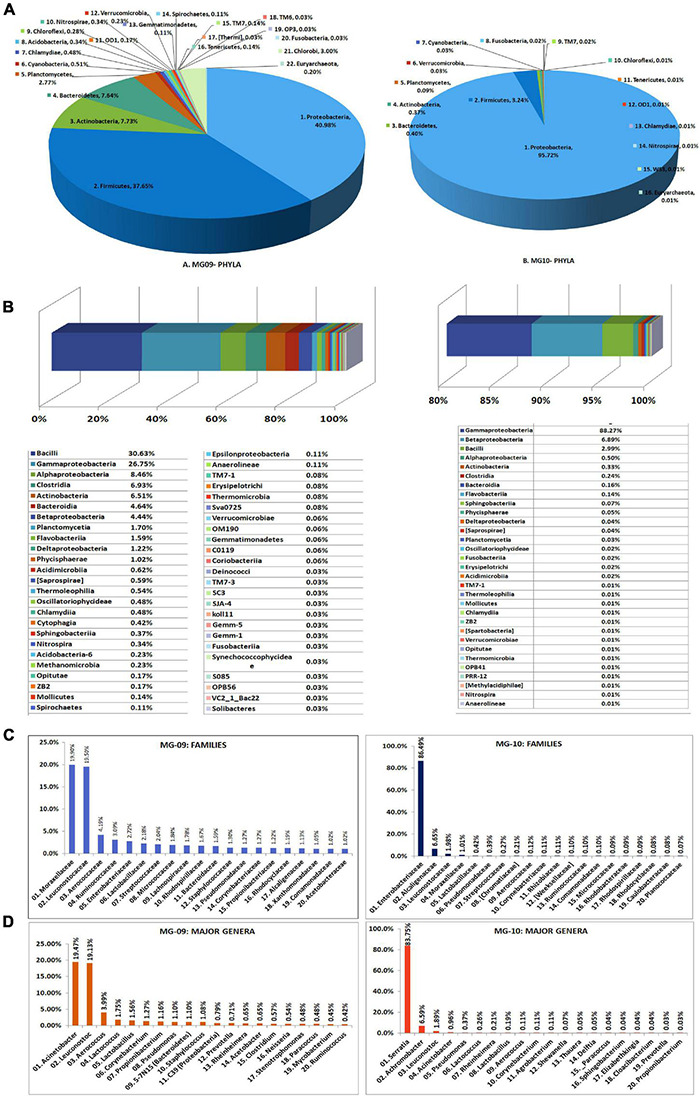
Illumina MiSeq NGS-based bacterial diversity analysis on watermelon seed-embryos with DNA extracted using PowerFood microbial DNA isolation kit (MG09) or Axygen DNA isolation kit (MG10). Distribution of phylogenetic groups based on 16S rRNA gene V3–V4 region taxonomic profiling at **(A)** phyla and **(B)** class levels. **(C)** Major families. **(D)** Major genera.

Seed-embryo homogenate pooled from five seed lots that were not showing any bacterial CFU during cultivation-based monitoring were employed in this study. The sample showed relatively low DNA yields with the two kits (10.5 ng μl^–1^ for PF kit and 7.26 ng μl^–1^ for AP kit.), but both (MG09 and MG10, respectively) gave rise to good 16S V3–V4 amplicon libraries (Supplementary Table 2). A large share of NGS-derived OTUs as per QIIME round-1 analysis corresponded to Cyanobacteria/chloroplast or Proteobacteria/mitochondria leaving a smaller fraction attributable to bacteria. QIIME round-2 analysis after filtering out the plant sequences showed a high bacterial diversity influenced by the DNA isolation kit (22 and 16 phyla, respectively, for MG09 and MG10) with the dominance of Proteobacteria (41.0% and 95.7%, respectively) followed by Firmicutes (37.6% and 3.2%, respectively). The former also displayed small shares of Actinobacteria, Bacteroidetes, and Planctomycetes along with minor shares of 17 other phyla including several candidate phyla and some Euryarchaeota (0.2%), with the latter displaying much less diversity (Figure 4A).

At the Class level, MG09 and MG10 showed 49 and 31 constituents, respectively, with 27 taxa common to both (Figure 4B). Although Bacilli formed the major class in MG09 (30.6%), this branched to the families Leuconostocaceae, Aerococcaceae, Lactobacillaceae, and Streptococcaceae. Gamma-Proteobacteria formed the next major class (26.7%) followed by Alphaproteobacteria, Clostridia, Actinobacteria, Bacteroidia, and Betaproteobacteria. MG10, on the other hand, had nearly 88% OTUs under γ-Proteobacteria followed by β-Proteobacteria and Bacilli. At the Family level, MG09 showed 166 constituents with Moraxellaceae and Leuconostocaceae forming the largest families (Figure 4C). MG10 showed about 92 families with the dominance of Enterobacteriaceae. At the Genus level, MG09 comprised 244 constituents with 123 defined genera while MG10 bore 129 constituents comprising of 72 defined genera. The distribution of top 25 defined families and genera indicated that the high shares of Proteobacteria and Firmicutes in MG09 was primarily ascribable to *Acinetobacter* and *Leuconostoc* spp., whereas MG10 had mostly *Serratia* sp. (Figure 4D). The results in one way reflected a significant effect due to the DNA extraction kit on bacterial diversity but, on the other hand, emphasized the need for different DNA extraction approaches to bring out a larger diversity from phyla to genera levels (Supplementary Dataset 2). Spore-forming bacteria constituted a negligible fraction in both MG09 (*Lactobacillus* 1.56%, *Bacillus* 0.23%, *Marinilactibacillus* 0.20%; *Paenibacillus* 0.06%; *Lysinibacillus* 0.03%) and MG10 (*Lactobacillus* 0.19%; *Bacillus* 0.03%).

### Whole Genome Metagenome Mediated Taxonomic and Functional Analyses on Watermelon Seed Embryos

The whole embryo homogenate used for DNA extraction gave better yields (24.4 ng μl^–1^) than in the earlier instance of using the supernatant fraction for the MG09 sample using the same PF DNA isolation kit. Illumina HiSeq yielded 13 million raw reads with 0.375 million contigs available for downstream analysis and 0.3 million contigs aligning to bacteria (Supplementary Table 3).

**FIGURE 5:**
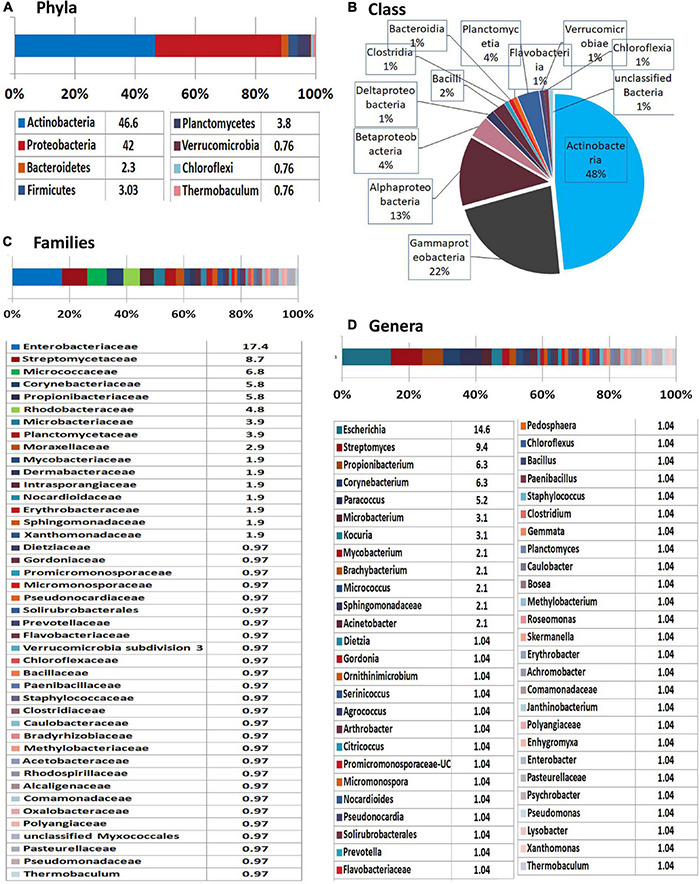
Illumina HiSeq NGS based whole genome metagenomics on watermelon seed-embryos. MEGAN-based taxonomic profiling of bacterial microbiome at the **(A)** phyla, **(B)** class, **(C)** family, and **(D)** genera levels.

MEGAN-mediated taxonomic profiling showed relatively less taxonomic diversity compared with the previous 16S rRNA gene V3–V4 profiling and a different taxonomic profile. Actinobacteria formed the most dominant phylum (47%) closely followed by Proteobacteria (42%) and then smaller shares of Planctomycetes (4%), Firmicutes (3%), and Bacteroidetes (2%) with the traces of Verrucomicrobia, Chloroflexi, and Thermobaculum (Figure 5). At the Class level, Actinobacteria formed the major constituent followed by γ-Proteobacteria, α-Proteobacteria, and β-Proteobacteria with Enterobacteriaceae constituting the dominant family. Spore-formers (*Bacillus* and *Paenibacillus* spp.) constituted a very minor share of 2% OTUs. Besides bringing out the prevalence of an array of diverse bacteria inside the embryos, the results endorsed the vertical transmission hypothesis in tune with the intracellular colonization.

**FIGURE 6:**
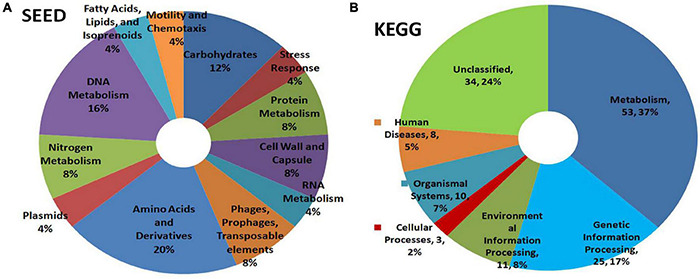
Illumina HiSeq NGS based whole genome metagenomics on watermelon seed-embryo bacterial microbiome functional profiling as per **(A)** SEED and **(B)** KEGG analyses.

SEED analysis of watermelon WMG data showed metabolism as the main functional role for the bacterial community covering amino acid, DNA, carbohydrate, nitrogen, protein, and RNA with other attributes including cell wall, stress response, motility, and others (Figure 6A). KEGG analysis also showed metabolism as the key function followed by genetic information processing, environmental information processing, and others with a major share (24%) remaining unclassified (Figure 6B).

### Cultivation- and Microscopy-Based Testing of Grapevine Seeds and Seed-Embryos for Endophytic Bacteria

**FIGURE 7:**
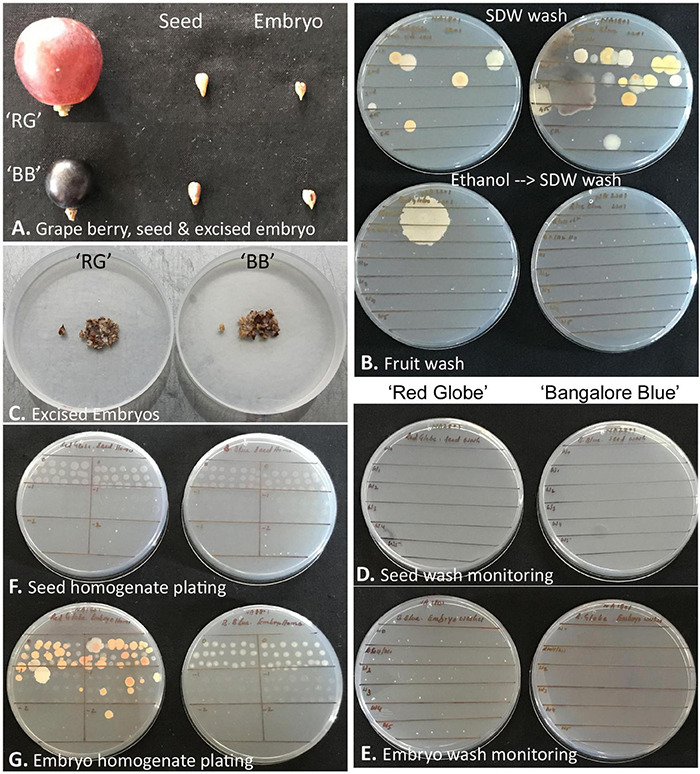
Description of grapevine seed *versus* excised seed-embryo 16S rRNA V3–V4 amplicon-based taxonomic profiling. **(A)** Berry, seed, and excised seed-embryo in ‘Red Globe’ (‘RG’) and ‘Bangalore Blue’ (‘BB’). **(B)** Monitoring the fruit wash solutions for the microbial load by spotting on nutrient agar, the upper panel showing results after six sterile distilled water (SDW) washes, and the lower panel showing the outcome after one ethanol wash followed by six FDW washes. **(C)** Excised embryos from the seeds of ‘RG’ and ‘BB.’ **(D)** Monitoring seed-wash solutions. **(E)** Monitoring embryo-wash solutions. **(F)** Testing the seed-tissue homogenate for cultivable bacteria through SP-SDS of serial dilutions with no bacterial CFU for 2–4 weeks. **(G)** Seed-embryo homogenate tested for cultivable bacteria through SP-SDS showing bacterial CFU after 2–4 weeks.

‘Red Globe’ had large bold berries compared with ‘Bangalore Blue,’ but the seed and embryo sizes were comparable for both grape varieties (Figure 7). Monitoring the berry wash solutions by spotting on NA showed a few colonies initially, but none after the ethanol step. Tracking the seed- and embryo-wash solutions did not show any bacteria in spotting tests or the plating of up to 400 μl of the last wash solution.

The whole seed tissue homogenates did not show any bacterial growth on NA or TSA for up to 2 weeks. Similarly, the embryo homogenates also did not display any microbial colonies for about 10 days, but thereafter, by 2–3 weeks, ‘RG’ homogenate showed multiple pigmented colonies (translated to about 1.0–2.5 × 10^3^ CFU g^–1^ tissue), while ‘BB’ occasionally showed a few white colonies from the thick homogenate applied spots of the original homogenate. Bright-field microscopy on seed and embryo homogenates, on the other hand, indicated abundant motile-bacterial cells which indicated that the CFU formed was far negligible relative to the cell numbers.

Three bacterial isolates were retrieved from ‘RG’ embryo-homogenate applied plates. Attempting 16S rRNA gene sequence-based bacterial identification, two isolates were established as *Roseomonas pecuniae* and *Kineococcus* sp. (Actinobacteria), while the third isolate turned out to be a mixture despite adopting three rounds of SP-SDS-mediated single-colony purification. All these isolates appeared short-lived failing to revive after 1-week storage under ambient-conditions or under refrigeration and, thus, could not be characterised. One isolate was obtained from ‘BB’ seed-embryo, which was identified as *Staphylococcus warneri* (Firmicutes).

### 16S V3–V4 Profiling of Seeds and Seed-Embryos in Grapevine

All the four samples, namely, ‘RG’ and ‘BB’ seeds and the excised seed-embryos, showed low DNA yields (3–5 ng μl^–1^), but they gave rise to good amplicon libraries with 0.23–0.27 million reads for seed samples and 0.19–0.31 million reads for seed-embryos (Supplementary Table 4). A major share of these reads, however, corresponded to chloroplast and mitochondria for the seed-embryos (36%–50%), but this formed only a minor share for whole seeds (1.2%–1.5%). The seed samples exhibited about 1800 OTUs, while the seed embryos displayed 840 OTUs excluding the plant reads. More than 99% of the OTUs corresponded to Eubacteria with a minor share of Archaea.

**FIGURE 8:**
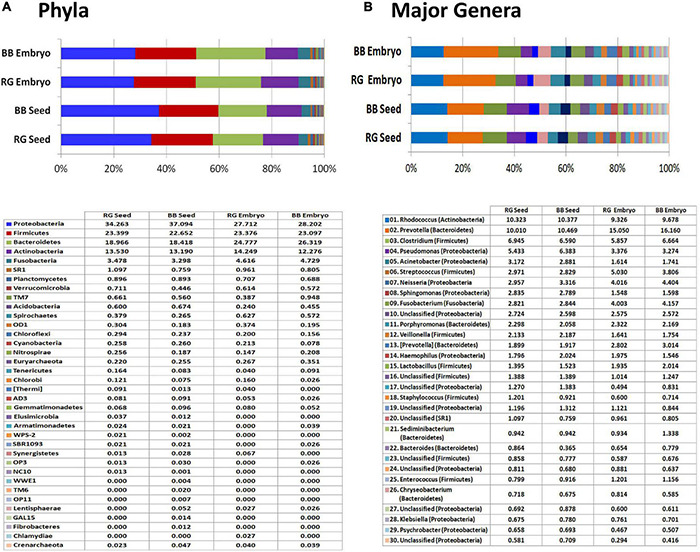
16S rRNA metagene V3–V4 region profiling with Illumina MiSeq NGS platform on grapevine seeds and embryos. **(A)** Distribution of OTUs (%) under different phyla. **(B)** Major genera (OTU %) in the four samples.

The read/OTU distribution for seed tissues showed a high bacterial diversity with 28 phyla for ‘RG’ and 34 for ‘BB’ with a more or less identical pattern for both the cultivars with respect to major phyla. Proteobacteria constituted about 35.7% share followed by Firmicutes (23%), Bacteroidetes (18.7%), Actinobacteria (13.4%), and Fusobacteria (3.4%). Other constituents included Planctomycetes, Verrucomicrobia, SR1, TM7, Acidobacteria, Spirochaetes, OD1, Chloroflexi, Cyanobacteria, Nitrospirae, Tenericutes, and Chlorobi, to name some (Figure 8A). Seed-embryos also displayed a high bacterial diversity with 24 phyla for both ‘RG’ and ‘BB’ and identical distribution pattern in respect of major phyla in both instances. Seed samples showed 28 phyla common to both which constituted 99.9%–100% OTUs, whereas the two embryo samples exhibited 21 phyla common to both cultivars (99.8%–99.9% OTUs) out of a total of 26 phyla documented. The main differences between the seed and embryo samples included relatively more diversity in the whole seeds (35 phyla) than in the embryos (30) considering both the cultivars, and lower levels of Proteobacteria and Bacteroidetes (phylum level) for embryos than whole seeds. One Archaeal phylum (Crenarchaeota) was also documented across the four samples although in minor shares.

At the Class level, a high amount of endophytic bacterial diversity was documented with 108 constituents in seed samples and 71 for embryos, with 10–12 phyla accounting for nearly 95% OTUs. The major ones across the four tissue samples included γ-proteobacteria, Bacteroidia, Clostridia, Actinobacteria, Bacilli, α-proteobacteria, β-proteobacteria, Fusobacteriia, and Flavobacteria (data not shown). While the seeds harbored more of γ-Proteobacteria and α-Proteobacteria which probably arose from the seed-coat, seed-embryos showed more of Bacteroidia, Bacilli, and Fusobacteriia. Family level distribution endorsed the prevalence of a high amount of bacterial diversity spanning over 330 constituents across the four samples. Embryos showed a high share of Prevotellaceae (15%–16%) than seeds (10%–10.5%) followed by Nocardiaceae (9.3%–10.4%), Clostridiaceae, Pseudomonadaceae, Moraxellaceae, Neisseriaceae, Streptococcaceae, and others.

Genus level distribution brought to light an unprecedented taxonomic diversity prevailing in embryos to the tune of about 350 genera (293 in ‘RG’ and 279 in ‘BB’) and about 570 genera for whole seeds (443 in ‘RG’ and 465 in ‘BB’) (Supplementary Dataset 3). The embryos showed *Prevotella* as the most dominant constituent (15.1% in ‘RG’ and 16.1% in ‘BB’) followed by *Rhodococcus* (9.3%–9.7%), *Clostridium, Streptococcus, Neisseria, Fusobacterium, Pseudomonas, Porphyromonas, [Prevotella]*, and *Lactobacillus*; the seeds showed similar amounts of *Prevotella* and *Rhodococcus* (10.0%–10.5%) followed by *Clostridium, Pseudomonas, Acinetobacter, Streptococcus, Neisseria, Sphingomonas, Fusobacterium, Porphyromonas*, and *Veillonella* (Figure 8B). Thus, a high diversity of seed-associated and vertically transmissible endophytic bacteria were documented but sharing similar taxonomic profiles for the two cultivars which were growing in two different parts of the world.

## Discussion

The study elucidates intracellular bacteria ‘Cytobacts’ as a ubiquitous phenomenon across vascular plants enduring perennially through embryo-mediated vertical transmission with considerable structural, functional, and genomic significance. As per the preexisting information, all plant species and plant organs are known to harbor endophytic bacteria, and substantial information has been emerging on the utility and application of such plant intimately associated microorganisms in crop husbandry toward plant growth promotion, bio-control of pathogens and pests, alleviation of abiotic stress, etc. (Hardoim et al., 2015; Liu et al., 2017; Araujo et al., 2020; Vandana et al., 2021). Initial studies on bacterial endophytes had adopted cultivation-based approaches for assessing the organismal diversity and toward the exploitation of beneficial organisms in agriculture and allied sectors (Hallmann et al., 1997; Rosenblueth and Martínez-Romero, 2006). Such studies have often brought out notable bacterial diversity in root tissues. Conversely, the shoot portions were not investigated proportionately or often showed relatively low CFU and less taxonomic diversity. Cultivation-independent molecular studies such as metagenomics or 16S rRNA gene amplicon profiling have revealed high amounts of bacterial taxonomic and functional diversity for root tissues (Bulgarelli et al., 2012; Sessitsch et al., 2012). Similar studies targeting shoot tissues, although relatively limited, have given a similar picture revealing a vast bacterial diversity (Dos-Santos et al., 2017; Thomas and Sekhar, 2017).

While cultivation-based and molecular-based explorations bring out the diversity data, they do not provide information about the abundance of microorganisms and the niches of colonization which are attained through microscopy. Although a number of publications on endophytic microorganisms are available giving the impression about such associations as ubiquitous even to the extent of stating that microbe-free plants are rather impossible (Partida-Martinez and Heil, 2011; Heil, 2015), they do not make mention about visualizing them inside the tissues. Studies giving microscopic evidence on tissue colonization by bacterial endophytes have often encompassed on root tissues showing both intercellular and intracellular colonization (Compant et al., 2005, 2008; White et al., 2014, 2018; Shehata et al., 2017). Thus, the current understanding suggests bacterial entry through root hairs or other root openings, traversing the root cortex with intracellular accommodation, xylem immigration, and subsequent vascular transmittance (Compant et al., 2011, 2021; Prieto et al., 2011). Microscopic studies on shoot tissue colonization by bacterial endophytes have been very few considering the large volume of literature on endophytic bacteria, which often give the impression of intercellular colonization with occasional reference to intracellular presence (Compant et al., 2005, 2008, 2011). This statement does not cover the nitrogen fixing genera which show intracellular colonization in root nodules. The general understanding about endophytic bacterial association in shoot tissues is that they are apoplastic or intercellular colonizers comprising of xylem, intercellular spaces, and vascular interconnections (Hallmann et al., 1997; Sattelmacher, 2001; Hardoim et al., 2015; Liu et al., 2017). On the other hand, some dedicated studies targeting the shoot system have shown intracellular colonization in field plants (Pirttilä et al., 2000; Thomas and Reddy, 2013; Koskimäki et al., 2015; Thomas et al., 2019) and also in tissue culture systems which are established from surface-sterilized tissues (de Almeida et al., 2009; Thomas and Sekhar, 2014; Esposito-Polesi et al., 2017).

Despite these established reports on intracellular bacterial colonization, which may constitute a negligible share considering the whole gamut of endophytic microbiome research, there seems to be not much research exploring deeper into tissue colonization aspects and clarifying the intercellular *versus* intracellular nature of associations which has significant implications for plant biology. A clearer picture on endophytic bacteria as intracellular inhabitants in healthy plant cells has emerged with the recent microscopic explorations of long-term actively maintained or freshly established, supposedly aseptic and axenic tissue cultures of grapevine, periwinkle, tobacco, *Arabidopsis*, and certain medicinal plant species. This showed the prevalence of an enormity of ‘Cytobacts’ in terms of both abundance and taxonomic diversity in cell and callus cultures (Thomas and Franco, 2021). The organisms proved to be largely uncultivable although a micro-share of such organisms could be activated to cultivation with specific treatments such as the use of host-tissue-extract in a low nutrient environment. *In vitro* activation of CREB is also a common feature in plant tissue cultures which generally emerge as microbial contaminations (Shaik and Thomas, 2019; Thomas et al., 2019). The present study brings to light ‘Cytobacts’ as a ubiquitous entity in vascular plants.

Intracellular presence of diverse ‘Cytobacts’ in shoot-tip tissues makes us infer that these organisms would move to the daughter cells during mitosis and thus reach all plant tissues. This also implies that there is possible gametic transmission through meiosis that brings in vertical movement to the next generation. This is evident from the molecular explorations reported in this study on watermelon and grape seed-embryos. Recent reports on endophytic bacteria in pollen grains (Maniranjan et al., 2017), excised seed-embryos in wheat (Kuźniar et al., 2020a,b), and in the *in vitro* raised tomato seedlings after seed-coat removal post-seed germination illustrate this significant result (Shaik and Thomas, 2019). Very recent studies employing excised seed-embryos, different parts of embryos, and embryo-derived *in vitro* seedlings of watermelon have clearly established vertical transmission of abundant and diverse bacteria prevailing as CREBs (Thomas and Sahu, 2021). Intracellular colonization facilitates the diverse organisms to reach the embryo and in turn all the cells of the zygote-derived new plant allowing a concoction of the organisms from either parent. Microbial association with the seed as determinants of seed quality, seedling vigor, and even in conferring disease resistance is greatly appreciated in plant biology (Nelson, 2018; Matsumoto et al., 2021). The observation on embryo-colonization by abundant and diverse bacteria adds a new insight in plant microbiology at variance from the general perception that endophytes are acquired by plants primarily from soil through roots, and from atmosphere through natural openings and other vectors (Frank et al., 2017; Kandel et al., 2017).

The endophytic bacterial diversity documented for shoot-tip tissues and seed-embryos in this study appeared quite vast, which included mainly Proteobacteria and varying shares of Firmicutes, Actinobacteria, and Bacteroidetes as the next major phyla and minor shares of other diverse phyla including several candidate phyla and some Euryarchaeota. It is to be borne in mind that orthodox seeds go through extreme drying which could affect the survival of the organisms. This necessitates the use of dried seeds to assess if the organisms survive the desiccation. Observations with watermelon seeds which were in storage for 6–12 months indicated the prevalence of high bacterial diversity as CREBs with the dominance of Firmicutes barring spore formers, with the activation of more organisms and a modified diversity profile in favor of Proteobacteria with seedling development (Thomas and Sahu, 2021). The extent of taxonomic diversity documented with the seed-embryos could be even more considering the effects due to the DNA extraction kit or the metagenomics approach on phylogenetic variability recorded in this study and in other reports. The bacterial diversity appeared to be masked initially with the predominance of chloroplast and mitochondrial sequences, removal of which helped in unraveling the phylogenetic variability as is the case in other cultivation-independent studies (Thomas and Sekhar, 2017; Thomas and Franco, 2021). Embryo excision from grape seeds warranted testa softening with concentrated alkali which additionally ensured the elimination of all seed external bacteria. It is worth noting that the two grape cultivars that were grown in two different continents shared identical taxonomic profiles for both seeds and seed-embryos. Seed/embryo colonization by endophytic bacteria has been demonstrated in *Vitis* sp. (Compant et al., 2011), *Cucumis melo* (Glassner et al., 2018), and in a few other instances (Berg and Raaijmakers, 2018; Nelson, 2018). The present study targeting embryo *per se* further strengthens such reports. A clear understanding of the cell constituents and their functioning is essential in plant cell biology. The elucidation of the ubiquitous existence of diverse microorganisms in live plant cells with their continuous vertical transmission opens up scope for further research on spin-off study areas in plant biology similar to the interdependent human- and animal-associated microbiota (Engel and Moran, 2013; Almeida et al., 2019). It might be possible to bring out more diversity adopting alternate methods to block the amplification of mitochondrial and plastid sequences, such as the design and the use of PNA PCR clamps (Lundberg et al., 2013; Lefèvre et al., 2020). This warrants a comparative study to assess if the two-step QIIME analysis adopted in this study, or the use of PNA clamps harnesses more taxonomic diversity.

It is indeed inexplicable as to how such ubiquitous intracellular bacterial association of a wide array of prokaryotes in live plant cells went unnoticed by plant biologists and microbiologists. The present ‘discovery’ was facilitated with follow-up research on intracellular micro-particle motility with live-cell imaging. It is possible that such observations in the past were overlooked, possibly as Brownian motion (Brown, 1828), or micro-organelle movement, cytoplasmic streaming, etc., as discussed elsewhere (Thomas and Franco, 2021). This may also be confounded by the cultivation-recalcitrance of associated organisms. Large shares of environmental organisms are known to defy cultivation for lack of clear information about the cultivation requirements (Colwell, 2009) and on account of their smaller genomes (Kantor et al., 2013). Bacteria are known to occur in viable but non-culturable (VBNC) state and they could switch between VBNC and cultivable states, which is reported as a common feature with endophytic bacteria (Podolich et al., 2015; Thomas et al., 2019). It might not be an exaggeration to state that the CFU formed from the shoot-tip, seed- or embryo-homogenates during the cultivation-based part of this investigation constituted just one-millionth to one-billionth of motile micro-particles noticed in the microscopic fields that were construed as bacteria. It perhaps needs the intervention of researchers from molecular physics to unravel this mystery.

With their ubiquitous and integral cellular associations, ‘Cytobacts’ have significant functional roles in native plants such as improved plant fitness, growth promotion, activating the plant immune system, or governing the responses to biotic and abiotic stresses (Podolich et al., 2015; Thomas et al., 2017). PICRUSt functional analysis provided certain indications in regard to plant cell-energetics, plant defense, and phyto-physiology. Endophytic bacteria have been alluded to be involved in plant growth promotion, plant defense, biocontrol of pathogens/pests, bioremediation, and as sources of novel biomolecules (Conn et al., 2008; Hardoim et al., 2015; Podolich et al., 2015; Liu et al., 2017) and possibly as the plant immune system (Thomas et al., 2017). Several metabolic pathways that are observed in plants could be aided by these microorganisms (Holland, 1997; Thomas and Franco, 2021). It was also significant to document a notable share of Archaea in the shoot-tip tissues of field plants, particularly for maize and tomato under the phylum Euryarchaeota and the class Methanobacteria. Archaea has also been documented in seed-embryos of watermelon and grapes albeit in minor shares, but a more significant share (5%) was documented earlier with tomato seeds (Thomas and Shaik, 2020). Archaeome is now receiving increasing attention as plant and seed associated beneficial organisms (Taffner et al., 2018; Wassermann et al., 2019).

The endophytic microbiome within the plant holobiome in a holistic consideration of the plant and of hologenome in plant breeding and functional hologenomics is gaining significance now (Vandenkoornhuyse et al., 2015; Khare et al., 2018). Next-generation plant breeding strategies could be significantly linked to microbiome selection (Gopal and Gupta, 2016). Pollen-mediated microbial integration (Mitter et al., 2017) and seed-microbiome-targeted progeny selections (Matsumoto et al., 2021) are emerging plant breeding strategies. The endophytic microbiome perhaps needs to be viewed as a complex plant trait (Wagner et al., 2016). The unearthing of the ubiquitous existence of abundant and diverse bacteria in field plants and the long-term actively maintained live cell cultures (Thomas and Franco, 2021) with their integral and vertical transmission across generations has far-reaching implications opening the gateway for research on various interrelated aspects. Their functional roles in plants need more discussion as to how plants have accommodated such a huge variety of endophytic bacteria in the different phenology of plants. The observations also assume significance in environmental microbiology since the organisms invariably return to the environment at the termination of the life-term of plants/tissues.

The high amounts of taxonomic diversity and the organismal abundance also assume significance in genomics considering the possibility of co-extraction of microbial DNA with the plant DNA. For instance, 500 bacterial species with an average genome size of 5 Mb could constitute about 2,500 Mb genome data compared with a plant genome of ∼500 Mb (Thomas et al., 2017). However, the possibility of contamination of host genetic material by bacterial DNA appeared low considering the poor DNA recovery from bacterial cells. Further, a high share of 16S rRNA sequences corresponded to plant sequences as documented in other studies (Thomas et al., 2017, 2019). Such bacterial sequences are easily removed with bioinformatics tools during genome sequencing projects. However, it is not possible to exclude or ignore the functional contributions of these organisms in native plants and the *in vitro* cultures.

The current observations also assume evolutionary significance in the context of serial endosymbiont theory (Margulis and Bermudes, 1985; Margulis, 2004), reviewed in detail by Martin et al. (2015). Plastids and mitochondria are known to be of prokaryotic origin. The high share of 16S rRNA amplicon reads that matched Cyanobacteria at the phylum level (chloroplasts at the class level) and Proteobacteria at the phylum level (Rickettsiales at the order level and mitochondria at the family level) was a common observation for most of the plant samples. During the course of evolution, the functional autotrophic plant cells possibly emerged by the integration of free-living bacteria as mitochondria and photosynthetic cyanobacteria as chloroplasts that somehow survived the endocytosis into the cytoplasm in an amoeba-like eukaryotic protist, losing their independent nature and becoming cell organelles during the course of time. Chloroplasts and mitochondria are double-walled, containing their own circular DNA, as in the case of bacteria, along with their own transcriptional and translational machinery and self-replication. The long-term association of diverse organisms in the cytoplasmic niche could possibly pave the way for future plant evolutions.

This study targeted mainly Phanerogamae covering angiosperms and one representative of gymnosperms, namely, pine. Preliminary microscopic observations on members of Cryptogamae such as Thallophytes (algae, fungi), Bryophytes (mosses), and Pteridophytes (ferns) through bright-field microscopy showed abundant intracellular bacteria suggesting ‘Cytobacts’ as a ubiquitous phenomenon in the plant kingdom. Fungal–bacterial endosymbiosis has been well studied with different systems such as *Burkholderia* sp. and *Rhizopus microspores*, *Nostoc punctiforme*, and *Geosiphon pyriforme*, etc., which share a symbiotic relationship (Pawlowska et al., 2018; Bastías et al., 2020). Bacterial endosymbionts associated with insects has been well documented and has been a topic of extensive research where the associations ranged from obligate mutualism to facultative parasitism with a large taxonomic diversity (Moran and Baumann, 2000; Kikuchi, 2009). The term Cytobacts was coined to describe the abundant cytoplasmic bacteria first documented in banana (Thomas and Sekhar, 2014). Subsequent molecular diversity analyses indicated that the population could include Archaea as well. It warrants further microscopic explorations targeting Archaea to verify if they also are cytoplasmic inhabitants.

In conclusion, all plants and plant organs ubiquitously harbor endophytic bacteria, and the microscopic observations in this study on shoot-tip tissues and the mature seed-embryos leads to the conclusion that all vascular plants bear abundant and diverse intracellular bacteria, and their numbers far exceed the amount of host cells considering that each host cell harbors an abundant amount of Cytobacts. The observations on shoot tissue indicated that Cytobacts could be distributed to all daughter cells through mitosis and to the gametes through meiosis on account of their presence in the cytoplasm. Observations on mature seed embryos of representative plants indicate that the organisms are transmitted vertically to the next generation with a high amount of taxonomic diversity. This way, the endophytic bacterial continuity is maintained in seed-propagated as well as clonally perpetuated plants, making ‘Cytobacts’ an integral part of plant cell biology.

## Data Availability Statement

The datasets presented in this study can be found in online repositories. The names of the repository/repositories and accession number(s) can be found below: https://www.ncbi.nlm.nih.gov/genbank/ (SAMN18092256, SAMN18092258, SAMN18092402, SAMN18092404, SAMN12726312, SAMN12726313, SAMN17848617, SAMN17848618, SAMN17848891, and SAMN17848989).

## Author Contributions

PT and CF conceived the idea and co-wrote the manuscript. PT undertook the microscopic and genomics studies in discussion with TR. TR critically edited the manuscript. All authors approved the final version of the manuscript.

## Conflict of Interest

The commercial company TB-CCB was partly involved in the study design, data collection, analysis, interpretation, and the writing of this article through the affiliation of PT (who was the CEO of the company). PT was employed by company Thomas Biotech & Cytobacts Centre for Biosciences and the research outcome on endophytic microorganisms is being utilized in the commercial micropropagation industry by the company. The remaining authors declare that the research was conducted in the absence of any commercial or financial relationships that could be construed as a potential conflict of interest.

## Publisher’s Note

All claims expressed in this article are solely those of the authors and do not necessarily represent those of their affiliated organizations, or those of the publisher, the editors and the reviewers. Any product that may be evaluated in this article, or claim that may be made by its manufacturer, is not guaranteed or endorsed by the publisher.

## Footnotes

1
http://www.eurofinsgenomics.co.in

2
www.xcelrislabs.com

3
www.scigenom.com

## Supplementary Material

Supplementary Figure 1 Fresh tissue sections of tender shoot or petiole tissue from different plant species under bright-field (1000×). These tissue sections show no obvious intercellular spaces, or limited space between cells at the junction of multiple cells, where no bacterial cells are generally observed, but display abundant bacteria in the cytoplasmic niche. Bacterial cells (indicated by arrow-head) are better viewed with zoom-out option. **(A)** Banana corm tissue. **(B)** Banana pseudostem tissue. **(C)** Papaya flower stalk. **(D)** Okra petiole tissue. **(E)** Hibiscus petiole tissue. **(F)** Hydrangea petiole tissue.

Supplementary Figure 2 A view of watermelon fruit, seeds, and seed-embryos used for embryo-microbiome studies. **(A)** Cut fruit used as source of seeds. **(B)** Aseptically gathered seeds. **(C)** De-coated seeds. **(D)** Seed-embryos after surface sterilization.

Supplementary Figure 3 A view of the cultivation-based assessment of watermelon seed-embryos for endophytic bacteria by plating the embryo-homogenate at different concentrations documented after 2–3 weeks of plating. **(A)** Original homogenate at the rate of one seed-embryo of about 25 mg in 1 ml sterile water, **(B)** after 1:10 dilution, and **(C)** after 1:100 dilution. No cultivable bacteria were observed in most instances except for isolated cases 1–2 weeks after the plating.

## Abbreviations

CREB: cultivation-recalcitrant endophytic bacteria
FDW: filter-sterilized distilled water post-autoclaving
NA: nutrient agar
SDW: sterile distilled water
SATS: spotting-and-tilt-spreading
SP-SDS: single plate – serial dilution spotting
TH: tissue homogenate
TSA: trypticase soy agar.
