# Supplementary figures and images for: Cytobacts: Abundant and Diverse Vertically Seed-Transmitted Cultivation-Recalcitrant Intracellular Bacteria Ubiquitous to Vascular Plants
Source: Front Microbiol. 2022 Mar 7;13:806222. doi: 10.3389/fmicb.2022.806222 (PMC8967353; doi:10.3389/fmicb.2022.806222)

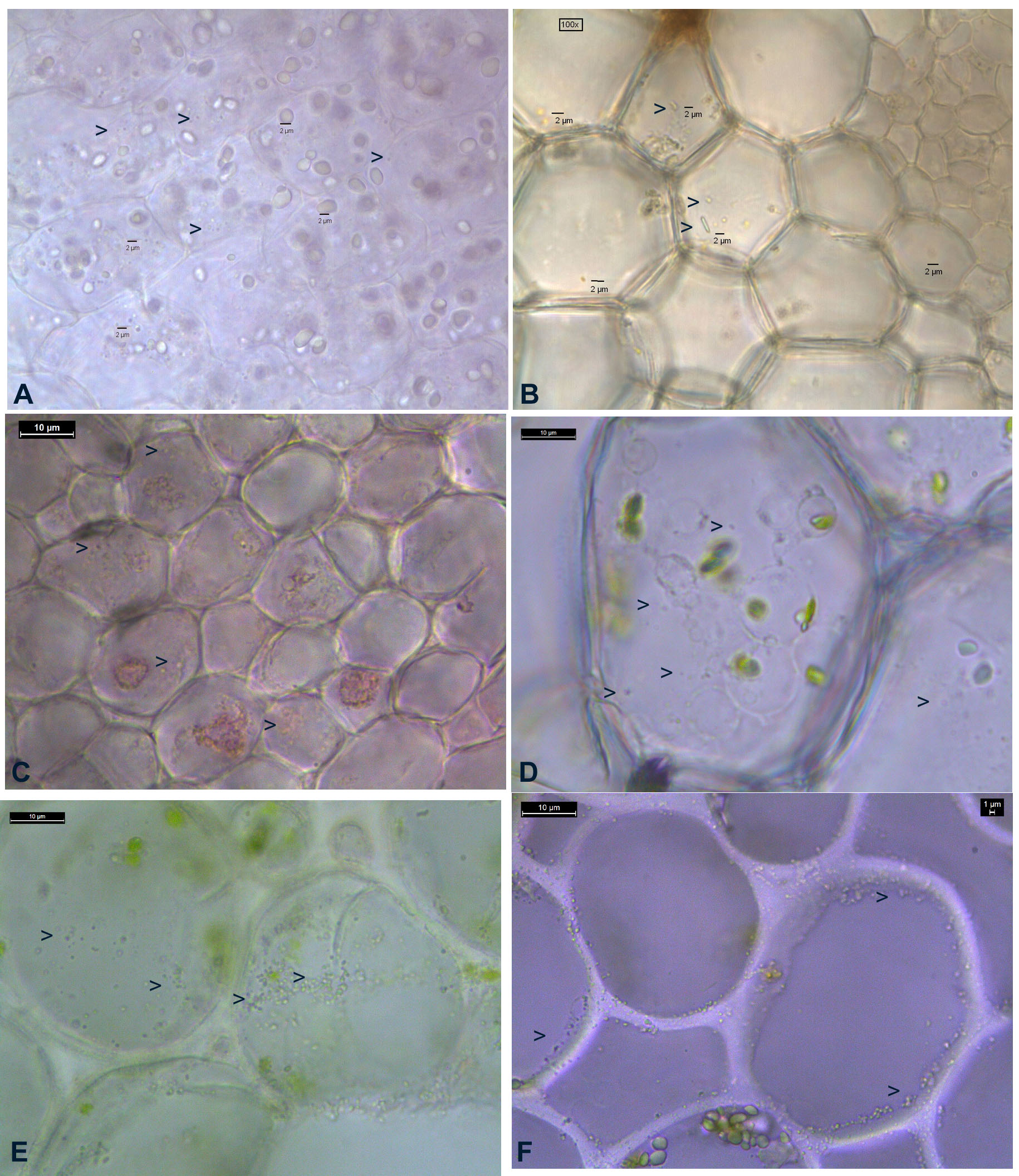

Supplement: Supplementary Figure 1 — Fresh tissue sections of tender shoot or petiole tissue from different plant species under bright-field (1000×). These tissue sections show no obvious intercellular spaces, or limited space between cells at the junction of multiple cells, where no bacterial cells are generally observed, but display abundant bacteria in the cytoplasmic niche. Bacterial cells (indicated by arrow-head) are better viewed with zoom-out option. (A) Banana corm tissue. (B) Banana pseudostem tissue. (C) Papaya flower stalk. (D) Okra petiole tissue. (E) Hibiscus petiole tissue. (F) Hydrangea petiole tissue. [file Image_1.JPEG]

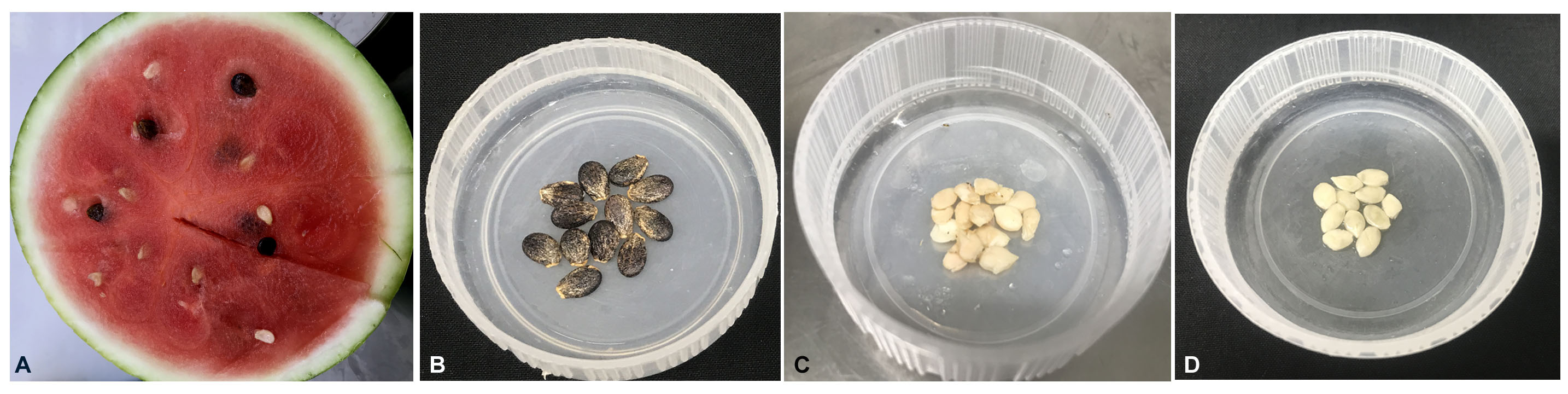

Supplement: Supplementary Figure 2 — A view of watermelon fruit, seeds, and seed-embryos used for embryo-microbiome studies. (A) Cut fruit used as source of seeds. (B) Aseptically gathered seeds. (C) De-coated seeds. (D) Seed-embryos after surface sterilization. [file Image_2.JPEG]

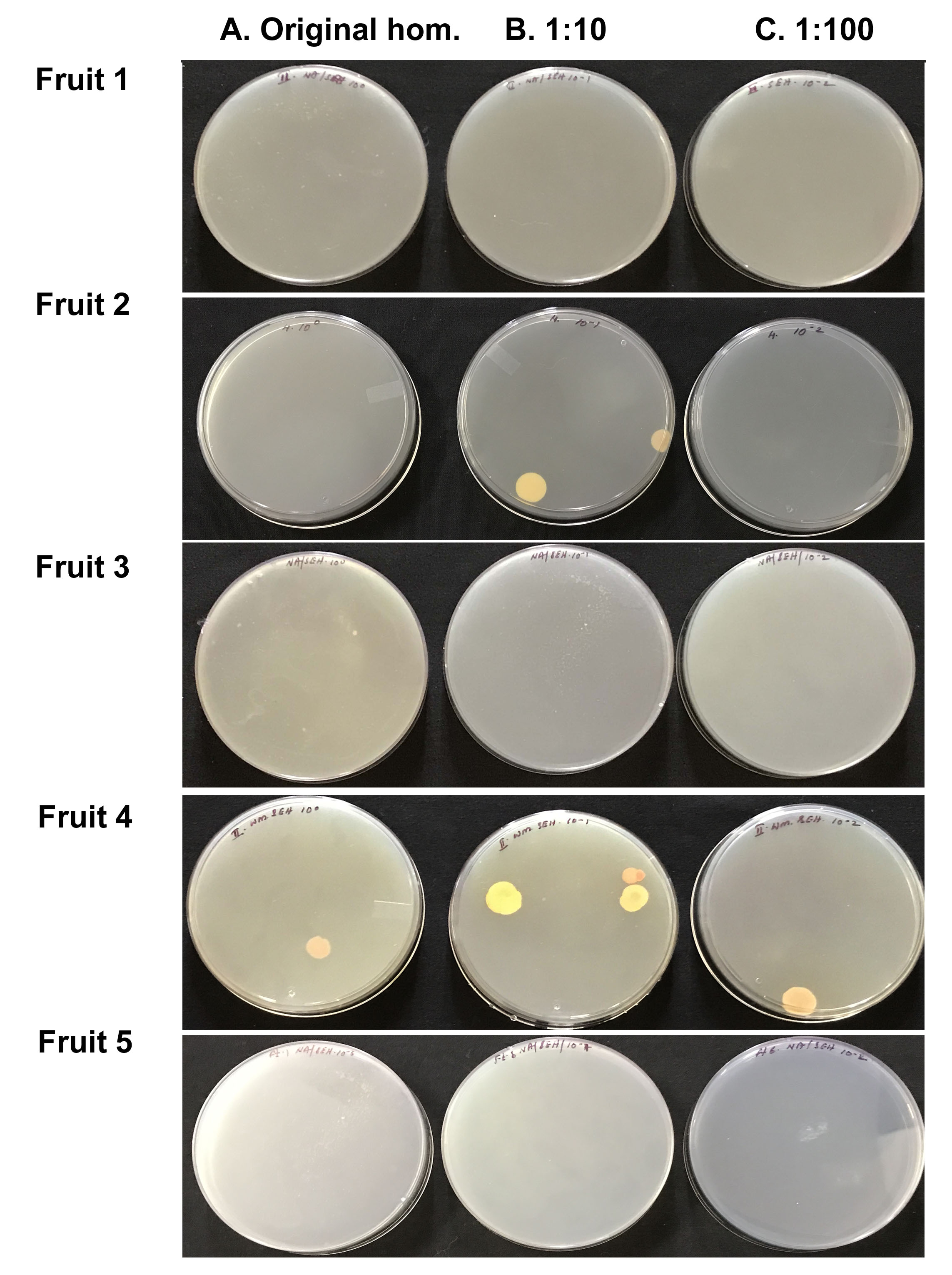

Supplement: Supplementary Figure 3 — A view of the cultivation-based assessment of watermelon seed-embryos for endophytic bacteria by plating the embryo-homogenate at different concentrations documented after 2–3 weeks of plating. (A) Original homogenate at the rate of one seed-embryo of about 25 mg in 1 ml sterile water, (B) after 1:10 dilution, and (C) after 1:100 dilution. No cultivable bacteria were observed in most instances except for isolated cases 1–2 weeks after the plating. [file Image_3.JPEG]
