# Supplementary material for: Cytobacts: Abundant and Diverse Vertically Seed-Transmitted Cultivation-Recalcitrant Intracellular Bacteria Ubiquitous to Vascular Plants
Source: Front Microbiol. 2022 Mar 7;13:806222. doi: 10.3389/fmicb.2022.806222 (PMC8967353; doi:10.3389/fmicb.2022.806222)
Supplement: Supplementary file 4 [file Table_1.DOCX]

**TABLE S1**. Data statistics on 16S rRNA V3-V4 amplicon profiling on four representative field plant shoot-tip tissues as per QIIME round-I analysis

|  | Particulars | **Plant species** | | | |
| --- | --- | --- | --- | --- | --- |
|  |  | Tomato | Watermelon | Periwinkle | Maize |
|  | NGS Sample ID | EG-Gen-MG01 | EG-Gen-MG02 | EG-Gen-MG03 | EG-Gen-MG04 |
|  | DNA yields (ng/ μl): Nanodrop | 11.0 | 9.1 | 8.5 | 7.3 |
|  | Total reads | 141,695 | 167,066 | 156,276 | 149,988 |
|  | Total bases | 69,738,775 | 86,590,609 | 80,418,504 | 74,137,151 |
|  | Data in Mb | ~70 | ~87 | ~81 | ~74 |
|  | **QIIME Round-I analysis** |  |  |  |  |
|  | Chloroplast Reads | 45,085 | 54,091 | 41,258 | 55,779 |
|  | Mitochondrial Reads | 34,864 | 38,898 | 31,150 | 38,032 |
|  | Chloroplast +Mtc reads | 79,949 | 92,989 | 72,408 | 93,811 |
|  | Chloroplast +Mtc reads: % reads | **56.4** | **55.7** | **46.3** | **62.6** |
|  | **QIIME Round-II analysis** |  |  |  |  |
|  | Reads |  |  |  |  |
|  | OTUs | 165 | 141 | 117 | 179 |
|  | Shannon alpha diversity | 2.538 | 2.042 | 3.463 | 3.801 |
|  | **OTU taxonomic assignment** | | | | |
|  | Eubacteria | 97.68 | 99.84 | 99.23 | 95.00 |
|  | Archaea | 2.32 | 0.16 | 0.77 | 5.00 |
