# Supplementary material for: Cytobacts: Abundant and Diverse Vertically Seed-Transmitted Cultivation-Recalcitrant Intracellular Bacteria Ubiquitous to Vascular Plants
Source: Front Microbiol. 2022 Mar 7;13:806222. doi: 10.3389/fmicb.2022.806222 (PMC8967353; doi:10.3389/fmicb.2022.806222)
Supplement: Supplementary file 5 [file Table_2.DOCX]

**TABLE S2.** Data Statistics on 16S rRNA V3-V4 amplicon profiling on watermelon seed embryo DNA extracted using PowerFood microbial DNA isolation kit (MG09) or Axygen DNA isolation kit (MG10) as per QIIME round-II analysis.

| Sample name | MG-09 | MG-10 |
| --- | --- | --- |
| DNA concentration (ug/ μl) | 10.5 | 7.26 |
| No. of reads | 830414 | 684190 |
| Total Data (Mb) | 394 | 341 |
| Mean Seq length | 265.8 | 283.7 |
| Guanine and Cytosine (GC) (%) | 55.0 | 48.0 |
| Stitch reads | 404931 | 413032 |
| Mean Seq length of Stitch Read | 449.9 | 460.4 |
| No. of reads after quality check | 404756 | 412780 |
| **QIIME Round - I analysis** |  |  |
| No. of OTUs | 1618 | 1481 |
| **Taxonomy at PHYLUM level (% OTUs)** |  |  |
| Cyanobacteria^†^ | 83.10 | 70.90 |
| Proteobacteria | 16.37 (16.0^‡^) | 28.50 (16.10^‡^) |
| Firmicutes | 0.33 | 0.40 |
| Actinobacteria | 0.07 | 0.00 |
| Bacteroidetes | 0.07 | 0.10 |
| Planctomycetes | 0.02 | **-** |
| **QIIME Round - II analysis** |  |  |
| Reads removed (assigned to chloroplast, mitochondria & unassigned) | 401220 (99.12%) | 359376  (87.1%) |
| **Total Data (Mb)** | 186 | 151 |
| No. of reads | 429194 | 324814 |
| No. of reads after QC | 33533 | 53404 |
| OTUs | 590 | 755 |
| Alpha diversity: Shannon index | 7.415979 | 1.684182 |
| Observed species | 619 | 798 |

^†^ turned out to be Chloroplast at class level; ^‡^mitochondria at family level
