# Supplementary material for: Cytobacts: Abundant and Diverse Vertically Seed-Transmitted Cultivation-Recalcitrant Intracellular Bacteria Ubiquitous to Vascular Plants
Source: Front Microbiol. 2022 Mar 7;13:806222. doi: 10.3389/fmicb.2022.806222 (PMC8967353; doi:10.3389/fmicb.2022.806222)
Supplement: Supplementary file 6 [file Table_3.DOCX]

**TABLE S3.** De novo whole genome metagenome data on taxonomic profiling of seed-embryo bacterial microbiome in watermelon (WMG 54) with Illumina HiSeq NGS platform

|  | Particulars | |
| --- | --- | --- |
| 1 | Sequencing Platform | Illumina HiSeq |
| 2 | Library type | Paired End (250bp × 2) |
| 3 | Project Type | *De novo* whole genome metagenomics |
| 4 | Total Reads | 13,480,926 |
| 5 | Sequence Length(bp) | 250 |
| 6 | Total Data (GB) | 6.74 |
| 7 | of GC | 39.72 |
| 8 | Average Base Quality | 37.34 |
| 9 | >=Q30 | 99.94 |
| 10 | Assembled Contigs: Total Contigs | 436,575 |
| 11 | Contigs <150 | 60,776 |
| 12 | Contigs 150-500 | 265,809 |
| 13 | Contigs >500 | 109,991 |
| 14 | Contigs >150 | 375,800 |
| 15 | Contigs corresponding to Viridiplantae | 16,508 (4.39) |
| 16 | Contigs selected for downstream analysis (for identifying bacteria) | 359,292 |
