# Supplementary material for: Cytobacts: Abundant and Diverse Vertically Seed-Transmitted Cultivation-Recalcitrant Intracellular Bacteria Ubiquitous to Vascular Plants
Source: Front Microbiol. 2022 Mar 7;13:806222. doi: 10.3389/fmicb.2022.806222 (PMC8967353; doi:10.3389/fmicb.2022.806222)
Supplement: Supplementary file 7 [file Table_4.DOCX]

**TABLE S4.** Data statistics on 16S rRNA V3-V4 amplicon profiling on grapevine fruit-derived seeds and the seed-embryos

|  | Particulars | Grapevine seeds | | Grapevine seed embryos | |
| --- | --- | --- | --- | --- | --- |
|  |  | Red Globe  (EG-Gr.MG01) | Bangalore Blue (EG-Gr.MG02) | Red Globe  (EG-Gr.MG03) | Bangalore Blue  (EG-Gr.MG04) |
|  | DNA yields (ng/ μl): Nanodrop | 5.3 | 3.2 | 4.2 | 3.0 |
|  | Total reads | 227,305 | 265,927 | 315,368 | 192,149 |
|  | Total bases | 108,452,759 | 126,725,808 | 100,386,815 | 88,069,126 |
|  | Data in Mb | ~108 | ~127 | ~100 | ~88 |
|  | **QIIME Round-I analysis** |  |  |  |  |
|  | Chloroplast Reads | 2,791 | 2,923 | 70,237 | 59,014 |
|  | Mitochondrial Reads | 525 | 258 | 44,693 | 36,496 |
|  | Chloroplast +Mtc reads | 3,316 | 3,181 | 114,930 | 95,510 |
|  | Chloroplast +Mtc: % reads | 1.46 | 1.20 | 36.44 | 49.70 |
|  | **QIIME Round-II analysis** |  |  |  |  |
|  | Reads | 223,989 | 262,746 | 200,438 | 96,639 |
|  | Observed OTUs | 1,814 | 1,869 | 840 | 840 |
|  | Shannon alpha diversity | 7.616 | 7.595 | 7.561 | 7.402 |
|  | **Taxonomic distribution** | | | | |
|  | Eubacteria | 99.78 | 99.74 | 99.65 | 99.73 |
|  | Archaea | 0.22 | 0.26 | 0.35 | 0.27 |
